# Supplementary material for: Complex intervention based on protective factors to improve resilience for gastric cancer patients: Mixed-methods process evaluation protocol
Source: PLoS One. 2025 Aug 13;20(8):e0329834. doi: 10.1371/journal.pone.0329834 (PMC12349701; doi:10.1371/journal.pone.0329834)
Supplement: S1 File — (DOCX) [file pone.0329834.s001.docx]

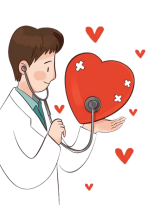

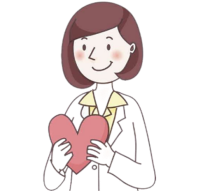


Your name：

Goal Achievement Log Book

Your goal：


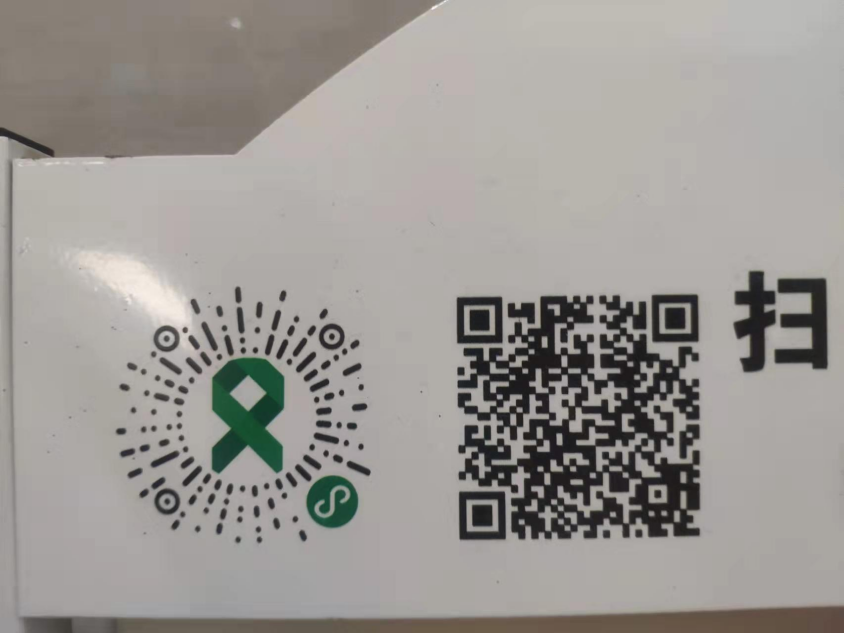


Good Doctor Consortium Patient Guide


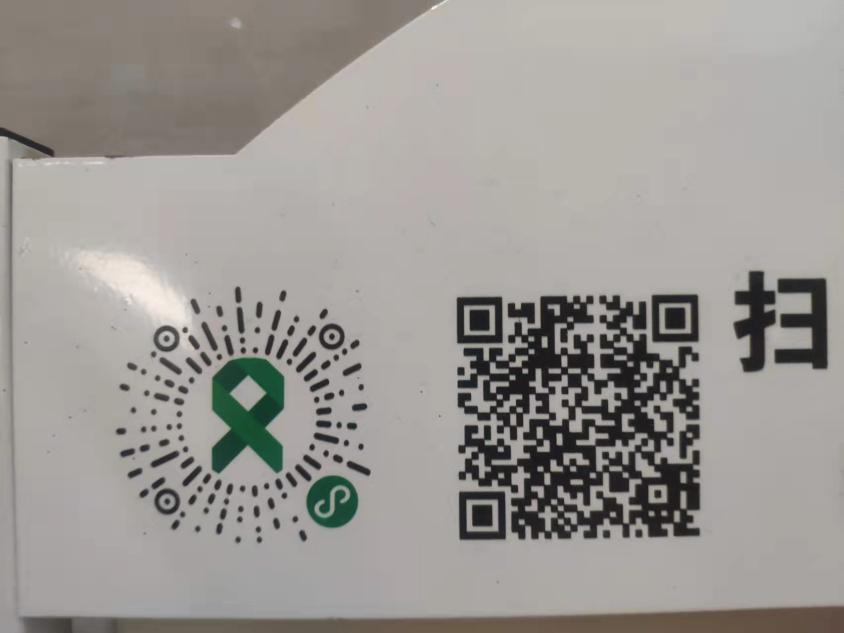


Good Doctor Consortium lite


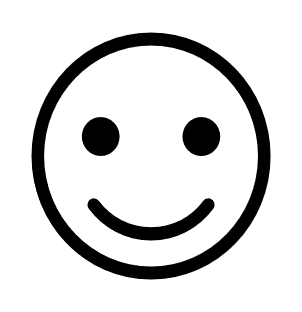


You can access relevant knowledge through the above-mentioned public account and mini-program.

| Date： No.____  稻壳儿宣传组  WPS金山防控中心 |
| --- |
| What is the theme of this session? ___________ |
| - Were you trained adequately for this session? ❏Yes  ❏No - Is this session proceeding as planned?❏Yes  ❏No - Have you fully understood and accepted this service?❏Yes  ❏No - Was the service tailored to your needs?❏Yes  ❏No - Are the service frequency and duration sufficient?❏Yes ❏No - Did you fulfill the researcher's tasks?❏Yes  ❏No - How long did it take to complete this service? minutes |
| What insights did you gain during the process of achieving this goal? |

Goal Achievement Log

| Date： No.____  稻壳儿宣传组  WPS金山防控中心 |
| --- |
| What is the theme of this session? ___________ |
| - Were you trained adequately for this session? ❏Yes  ❏No - Is this session proceeding as planned?❏Yes  ❏No - Have you fully understood and accepted this service?❏Yes  ❏No - Was the service tailored to your needs?❏Yes  ❏No - Are the service frequency and duration sufficient?❏Yes ❏No - Did you fulfill the researcher's tasks?❏Yes  ❏No - How long did it take to complete this service? minutes |
| What insights did you gain during the process of achieving this goal? |

Goal Achievement Log

| Date： No.____  稻壳儿宣传组  WPS金山防控中心 |
| --- |
| What is the theme of this session? ___________ |
| - Were you trained adequately for this session? ❏Yes  ❏No - Is this session proceeding as planned?❏Yes  ❏No - Have you fully understood and accepted this service?❏Yes  ❏No - Was the service tailored to your needs?❏Yes  ❏No - Are the service frequency and duration sufficient?❏Yes ❏No - Did you fulfill the researcher's tasks?❏Yes  ❏No - How long did it take to complete this service? minutes |
| What insights did you gain during the process of achieving this goal? |

Goal Achievement Log

| Date： No.____  稻壳儿宣传组  WPS金山防控中心 |
| --- |
| What is the theme of this session? ___________ |
| - Were you trained adequately for this session? ❏Yes  ❏No - Is this session proceeding as planned?❏Yes  ❏No - Have you fully understood and accepted this service?❏Yes  ❏No - Was the service tailored to your needs?❏Yes  ❏No - Are the service frequency and duration sufficient?❏Yes ❏No - Did you fulfill the researcher's tasks?❏Yes  ❏No - How long did it take to complete this service? minutes |
| What insights did you gain during the process of achieving this goal? |

Goal Achievement Log

| Date： No.____  稻壳儿宣传组  WPS金山防控中心 |
| --- |
| What is the theme of this session? ___________ |
| - Were you trained adequately for this session? ❏Yes  ❏No - Is this session proceeding as planned?❏Yes  ❏No - Have you fully understood and accepted this service?❏Yes  ❏No - Was the service tailored to your needs?❏Yes  ❏No - Are the service frequency and duration sufficient?❏Yes ❏No - Did you fulfill the researcher's tasks?❏Yes  ❏No - How long did it take to complete this service? minutes |
| What insights did you gain during the process of achieving this goal? |

Goal Achievement Log

| Date： No.____  稻壳儿宣传组  WPS金山防控中心 |
| --- |
| What is the theme of this session? ___________ |
| - Were you trained adequately for this session? ❏Yes  ❏No - Is this session proceeding as planned?❏Yes  ❏No - Have you fully understood and accepted this service?❏Yes  ❏No - Was the service tailored to your needs?❏Yes  ❏No - Are the service frequency and duration sufficient?❏Yes ❏No - Did you fulfill the researcher's tasks?❏Yes  ❏No - How long did it take to complete this service? minutes |
| What insights did you gain during the process of achieving this goal? |

Goal Achievement Log

Well done on goal!

Thanks for cooperation!


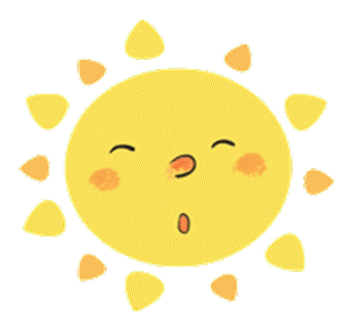


| **Home Care Tips (Scan QR code to view on mobile)** | |
| --- | --- |
| What to do about chemo nausea and vomiting? | 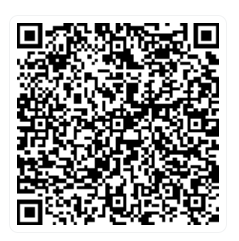 |
|  |  |
| What to do about chemo constipation? | 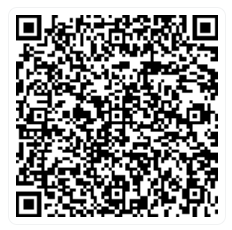 |
|  |  |
| If you're interested in exercising, check out the video on the right. | 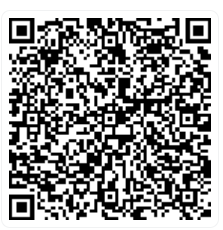 |
| What to eat and how to prepare it for stomach ailments? | 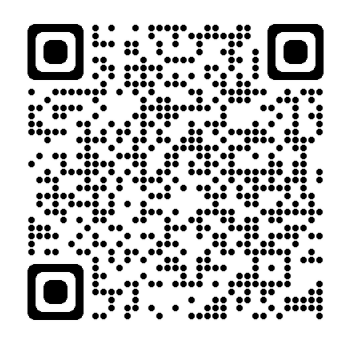 |
