# Supplementary material for: Complex intervention based on protective factors to improve resilience for gastric cancer patients: Mixed-methods process evaluation protocol
Source: PLoS One. 2025 Aug 13;20(8):e0329834. doi: 10.1371/journal.pone.0329834 (PMC12349701; doi:10.1371/journal.pone.0329834)
Supplement: S3 File — (DOCX) [file pone.0329834.s003.docx]

### Semi-structured interview guide

**Introduction:**

Dear Mr./Ms. [Interviewee's Name], thank you very much for taking the time to participate in our interview. We are conducting a study on resilience intervention for gastric cancer patients undergoing chemotherapy, based on protective factors. Your experiences and insights as a participant are extremely valuable to us.

Before we begin, I would like to briefly introduce our interview. We will ask for your opinions on this study and your experiences during the intervention implementation. Your feedback will help us better understand the factors influencing the implementation of the intervention. The interview will last between 30 to 60 minutes. Your responses will be recorded and transcribed to ensure accuracy in reflecting your thoughts. Please rest assured that our goal is to respect your time and privacy, and confidentiality of all information will be maintained.

Furthermore, I want to emphasize that there are no right or wrong answers – we simply want to hear your genuine thoughts and feelings. Throughout the interview, if you have any questions or need a break, please feel free to let me know.Now, if you are ready, we can begin. Once again, thank you for your participation and support.

**Intervention staff:**

1. How do you understand our intervention plan? *(intervention staff's perspectives on the intervention)*

2. How does this intervention align with our existing psychological care models and values? How does it fit with patients' values? How does it integrate into our routine psychological care processes and environment? *(inner setting - compatibility)*

3. In your opinion, what are the advantages of the intervention measures in this study compared to routine psychological care and other psychological care methods? *(intervention characteristics - relative advantage)*

4. How do you ensure that the intervention is implemented correctly and comprehensively? *(process - implementation)*

5. What challenges did you encounter during the implementation process? *(process - engaging)*

6. What do you perceive as the complexities in implementing the intervention? *(intervention characteristics - complexity)*

7. What efforts did you make to implement the intervention? Do you feel confident in implementing the intervention effectively? *(characteristics of individuals - capability)*

8. Can you tell me about your approach to building relationships with patients? *(inner setting - networks and communications)*

9. Could you describe efforts you made during past intervention implementations to better understand the needs of patients during the intervention process? *(inner setting - access to knowledge and information)*

10. What key partners does our organization have in advancing intervention implementation? How do these partnerships facilitate or hinder the adoption and implementation of the intervention? *(outer setting - partnerships and connections)*

11. Have there been pressures from other stakeholders and the work environment (clinical nurses)? How do they influence the implementation of the intervention? *(outer setting - external pressures)*

12. Is our intervention adaptable to different recipients and environments? *(intervention characteristics - adaptability)*

13. What improvements are needed for the intervention to better meet the needs of recipients and adapt to external implementation environments? *(process - adapting)*

14. How do we ensure the sustainability of the intervention? *(process - maintenance)*

15. Do you have any additional thoughts or experiences to share regarding the implementation of this intervention? *(process - reflecting and evaluating)*

**Participants:**

1. Could you please share your experience participating in this service activity?*(participants' experience of the intervention process)* Do you feel this psychological care arrangement meets your needs? *(characteristics of individuals - need)*

2. Were you actively involved in the intervention? How motivated were you to participate? *(characteristics of individuals - motivation)*

3. What motivated you to participate in this activity? Why did you join this activity? *(reach)*

4. How well does our psychological care align with your values and established beliefs? Are there any differences? *(inner setting - compatibility)*

5. In your opinion, what are the advantages of this new care approach compared to the psychological care or other psychological services you have received before? *(intervention characteristics - relative advantage)*

6. Do you find this new psychological care approach complex compared to previous experiences? *(intervention characteristics - complexity)*

7. What challenges did you encounter in completing the psychological care tasks? *(process - engaging)*

8. What efforts did you make to complete our tasks? Do you feel confident in your ability to complete tasks well? *(characteristics of individuals - capability)*

9. What adjustments did you make to meet our task requirements? *(intervention characteristics - adaptability)*

10. Did you face any external pressures or challenges while completing our task requirements? *(outer setting - external pressure)*

11. Do you believe the knowledge provided in our psychological care is what you need? Are there any knowledge areas we did not cover that you would find helpful? *(inner setting - access to knowledge and information)*

12. What were the most important activities you engaged in throughout the psychological care process? (Which part do you feel helped the most?) Were there any activities that were unnecessary or did not yield the expected results? *(participants' perspectives on the intervention)*

13. Do you think this type of psychological care needs adjustments based on your specific circumstances? *(process - adapting)*

14. What are your thoughts on our combined online and offline approach? *(inner setting - space)*

15. What impact did the psychological care service have on key outcomes? (e.g., impact on physical and mental states, or on family) *(efficacy)*

16. How did your physical and mental state fluctuate throughout the psychological care process? *(participants' experience of the intervention process)*

17. How did you maintain contact with our intervention personnel during the psychological care process? How was the relationship? *(outer setting - partnerships and connections)*

18. Are you still satisfied with our psychological care service? *(adoption)*

19. Do you think this psychological care service is necessary? (Is there a need for its existence and expansion?) Do you believe this psychological care service should continue to be provided? Will you continue the psychological exercise methods in the activities after the end? *(maintenance)*

20. Is there any challenge or experience we haven't covered that you would like to share with me? *(process - reflecting and evaluating)*

**The domains and content of the interview guide.**

| Interviewee | Target domain | Theoretical framework | Interview content |
| --- | --- | --- | --- |
| Intervention staff | Perspectives on the intervention |  | - How do you understand our intervention plan? |
|  | Impact factors of intervention implementation | CFIR-intervention characteristics | - In your opinion, what are the advantages of the intervention measures in this study compared to routine psychological care and other psychological care methods? - What do you perceive as the complexities in implementing the intervention? - Is our intervention adaptable to different recipients and environments? |
|  |  | CFIR-inner setting | - How does this intervention align with our existing psychological care models and values? How does it fit with patients' values? How does it integrate into our routine psychological care processes and environment? - Can you tell me about your approach to building relationships with patients? - Could you describe efforts you made during past intervention implementations to better understand the needs of patients during the intervention process? |
|  |  | CFIR-outer setting | - What key partners does our organization have in advancing intervention implementation? How do these partnerships facilitate or hinder the adoption and implementation of the intervention? - Have there been pressures from other stakeholders and the work environment (clinical nurses)? How do they influence the implementation of the intervention? |
|  |  | CFIR-characteristics of individuals | - What efforts did you make to implement the intervention? Do you feel confident in implementing the intervention effectively? |
|  |  | CFIR-process | - How do you ensure that the intervention is implemented correctly and comprehensively? - What challenges did you encounter during the implementation process? - What improvements are needed for the intervention to better meet the needs of recipients and adapt to external implementation environments? - How do we ensure the sustainability of the intervention? - Do you have any additional thoughts or experiences to share regarding the implementation of this intervention? |
| Participants | Perspectives on the intervention |  | - Could you please share your experience participating in this service activity? - What were the most important activities you engaged in throughout the psychological care process? (Which part do you feel helped the most?) Were there any activities that were unnecessary or did not yield the expected results? - How did your physical and mental state fluctuate throughout the psychological care process? |
|  | Reach | RE-AIM | - What motivated you to participate in this activity? Why did you join this activity? |
|  | Efficacy | RE-AIM | - What impact did the psychological care service have on key outcomes? (e.g., impact on physical and mental states, or on family) |
|  | Adoption | RE-AIM | - Are you still satisfied with our psychological care service? |
|  | Maintenance | RE-AIM | - Do you think this psychological care service is necessary? (Is there a need for its existence and expansion?) Do you believe this psychological care service should continue to be provided? Will you continue the psychological exercise methods in the activities after the end? |
|  | Impact factors of intervention implementation | CFIR-intervention characteristics | - In your opinion, what are the advantages of this new care approach compared to the psychological care or other psychological services you have received before? - Do you find this new psychological care approach complex compared to previous experiences? - What adjustments did you make to meet our task requirements? |
|  |  | CFIR-inner setting | - How well does our psychological care align with your values and established beliefs? Are there any differences? - Do you believe the knowledge provided in our psychological care is what you need? Are there any knowledge areas we did not cover that you would find helpful? - What are your thoughts on our combined online and offline approach? |
|  |  | CFIR-outer setting | - Did you face any external pressures or challenges while completing our task requirements? - How did you maintain contact with our intervention personnel during the psychological care process? How was the relationship? |
|  |  | CFIR-characteristics of individuals | - Do you feel this psychological care arrangement meets your needs? - Were you actively involved in the intervention? How motivated were you to participate? - What efforts did you make to complete our tasks? Do you feel confident in your ability to complete tasks well? |
|  |  | CFIR-process | - What challenges did you encounter in completing the psychological care tasks? - Do you think this type of psychological care needs adjustments based on your specific circumstances? - Is there any challenge or experience we haven't covered that you would like to share with me? |
