# Supplementary material for: Complex intervention based on protective factors to improve resilience for gastric cancer patients: Mixed-methods process evaluation protocol
Source: PLoS One. 2025 Aug 13;20(8):e0329834. doi: 10.1371/journal.pone.0329834 (PMC12349701; doi:10.1371/journal.pone.0329834)
Supplement: S4 File — (PDF) [file pone.0329834.s004.pdf]

Approved study protocol by ethics committee: 84230068

Ethical Review Application Form for the Biomedical Ethics Committee of Anhui Medical University

Key Points of the Research Proposal:

1.Research Background

Gastric cancer is the third most prevalent cancer in China, with chemotherapy serving as a primary postoperative adjuvant treatment. This treatment often causes significant psychological and symptomatic distress in patients. Resilience is a process where individuals under internal and external pressure tap into their cognitive abilities, capabilities, or psychological traits, and utilize internal and external resources to actively adjust and repair their mechanisms, focusing on positive psychological responses and the activation of internal potential in adversity. Therefore, intervention strategies aimed at enhancing patients' psychological resilience can improve their psychological coping skills, helping them use their own strengths to alleviate negative physical and psychological experiences.

Currently, research on resilience, both domestically and internationally, is still in the exploratory stage. Many resilience intervention programs lack a specific theoretical framework, are not based on evidence, and fail to consider the dynamic changes in resilience. Moreover, these interventions often overlook the interaction between multiple dimensions and their impact on patients' psychological resilience. In line with the "Healthy China 2023 Action Plan," this study, based on social-ecological systems theory and empirical research, explores comprehensive intervention strategies to enhance the psychological resilience of cancer survivors, thereby promoting their physical and mental health and improving their well-being.

The research team previously developed a resilience intervention program centered on protective factors, involving both patients and their families. Preliminary applications of this program have shown good feasibility, acceptability, and effectiveness. We now aim to conduct a large-scale clinical trial to further validate and refine the intervention program and to explore strategies for enhancing resilience through semi-structured interviews, ultimately summarizing the best evidence.

2. Research Objectives and Expected Outcomes

2.1 Research Objectives:

(1) To verify the effectiveness of a resilience intervention program based on protective factors for gastric cancer patients and conduct a process evaluation, dynamically assessing issues within the intervention program, refining and revising the program, and providing evidence for enhancing cancer survivors' resilience.

(2) To explore, through qualitative research, the experiences, opinions, and attitudes of cancer survivors and their stakeholders towards strategies for enhancing

resilience, providing references for strategy development.

(3) To summarize the best evidence for resilience enhancement strategies for cancer survivors, guiding the revision of existing strategies and the proposal of new ones to better achieve the goal of improving resilience in cancer survivors.

2.2 Expected Outcomes:

Completion of the research project, resulting in the publication of 1-2 papers.

3. Research Design and Methods

This study focuses on gastric cancer patients undergoing chemotherapy as the representative sample.

3.1 Empirical Research:

3.1.1 Research Setting: Oncology Department of a tertiary hospital in Anhui Province.

3.1.2 Research Period: From July 1, 2023, to June 30, 2026.

3.1.3 Participants:

Inclusion Criteria:

(1) Patients diagnosed with gastric cancer through pathological examination.

(2) Age ≥18 years.

(3) Patients who have undergone their first gastric cancer surgery and plan to undergo chemotherapy at the research hospital, with follow-up conditions.

(4) Patients whose condition allows for participation, who have provided informed consent, and are cooperative.

(5) Patients with an expected survival period of more than six months.

Exclusion Criteria:

(1) Patients with communication and understanding impairments.

(2) Patients with other severe diseases.

(3) Patients with a history of psychological disorders or mental illness.

(4) Patients currently participating in other clinical studies.

Dropout Criteria: Patients who, for various reasons, do not complete the entire resilience intervention program.

3.1.4 Sampling Method: Convenience sampling.

3.1.5 Sample Size Calculation: Sample size was calculated using G*Power software. Based on the pilot study results and the estimated effect size for the primary outcome measure, psychological resilience, and accounting for a 10% dropout rate, the final sample size is determined to be 196 participants, with 98 in the intervention group and 98 in the control group.

3.1.6 Data Management and Analysis: Data will be double-entered using Epidata 3.1, and analyzed using IBM SPSS 24.0 software. Statistical significance is set at $P \leq 0.05$. Quantitative variables with normal distribution will be compared using independent sample t-tests, while non-normally distributed variables will be analyzed using the Wilcoxon rank-sum test. Qualitative variables will be compared using the chi-square test.

3.2 Qualitative Research:

3.2.1 Participants:

Participants will be purposively sampled from the intervention group of the

empirical study, following the principle of maximum variation, selecting patients with diverse ages, educational backgrounds, occupations, and exercise habits. The sample size will be determined by data saturation, i.e., when no new themes emerge.

3.2.2 Data Collection and Analysis:

Semi-structured interviews will be conducted after informing the participants about the purpose and methods of the interviews and obtaining their informed consent. Interviews will be recorded, anonymized, and transcribed verbatim. Data will be organized and analyzed using Nvivo 11.0 software, with statistical descriptions of participants' sociodemographic characteristics and thematic analysis of interview data using Colaizzi's seven-step phenomenological method.

3.3 Evidence Summary:

3.3.1 Literature Sources:

The study will retrieve evidence on resilience enhancement in cancer patients from databases including BMJ Best Practice, UpToDate, Cochrane Library, JBI Evidence-Based Healthcare Center, International Guideline Network, Scottish Intercollegiate Guidelines Network, National Comprehensive Cancer Network, Registered Nurses' Association of Ontario, European Society for Medical Oncology, American Society of Clinical Oncology, Italian Society of Medical Oncology, PubMed, Web of Science, and Chinese databases such as CNKI, Wanfang Data, VIP, and Chinese Biomedical Literature Database. The search will cover literature published up to December 2023, with core search terms including "cancer," "resilience," and their synonyms or variations.

3.3.2 Research Methods:

Different quality assessment methods will be applied based on the type of research included. The quality assessment process will be independently conducted by two researchers, and any discrepancies will be resolved through consultation with a third party until consensus is reached.

4. Study Implementation Process

Flowchart:

5. Risk/Benefit Analysis

Benefits:

This study aims to enhance the psychological resilience of patients, improving their quality of life. If successfully conducted, the study can provide valuable insights for developing resilience intervention programs for other chronic disease patients.

Risks:

For patients, this study involves a non-invasive resilience intervention for eligible participants. Professional healthcare personnel will provide care during the intervention, and patients can withdraw at any time during the study. All researchers have undergone standardized training.

6. Participant Recruitment and Protection Measures

6.1 Recruitment:

Inclusion Criteria:

(1) Patients diagnosed with gastric cancer through pathological examination.

(2) Age ≥18 years.

(3) Patients who have undergone their first gastric cancer surgery and plan to undergo chemotherapy at the research hospital, with follow-up conditions.

(4) Patients whose condition allows for participation, who have provided informed consent, and are cooperative.

(5) Patients with an expected survival period of more than six months.

Exclusion Criteria:

(1) Patients with communication and understanding impairments.

(2) Patients with other severe diseases.

(3) Patients with a history of psychological disorders or mental illness.

(4) Patients currently participating in other clinical studies.

Dropout Criteria: Patients who, for various reasons, do not complete the entire resilience intervention program.

6.2 Protection Measures:

Participants will be informed of all relevant information about the study and its progress, allowing them to decide voluntarily whether to continue participating. Participants may withdraw from the study at any time without affecting their medical care or rights. If continuing in the study poses a significant risk, the study will be terminated. During the study, participants can report any discomfort to the research team.

7. Informed Consent Process

You are invited to participate in a study led by Zhao Xia from the School of Nursing at Anhui Medical University. This study, conducted from August 2023 to December 2024, aims to assess the application effectiveness of a resilience intervention program for gastric cancer patients undergoing chemotherapy. You have been invited to participate because you meet the study's criteria. The research team will fully explain the content of the informed consent form to you. Please carefully read the form and make a thoughtful decision about whether to participate. If you are currently enrolled in another study, please inform the research team. During the study, you can access information about the study and its progress at any time and decide voluntarily whether to continue participating. If you experience any discomfort during the study, please inform the research team.

8. Costs, Compensation, and Potential Compensation for Study-Related Harm

All technical and material costs associated with this clinical study (including material printing costs) will be covered by the project funds. There will be no additional financial burden on the participants. During the study, small gifts may be provided to participants as a reward, funded by the project. The patients and their families are the direct beneficiaries.

9. Data Collection, Management, and Quality Control

Data Management and Analysis:

Data will be double-entered using Epidata

Informed Consent Form

Introduction:

You are being invited to participate in a research study led by Professor Zhang

Xinqiong from the School of Nursing at Anhui Medical University. This study aims to evaluate the effectiveness of a psychological resilience intervention program for gastric cancer patients undergoing chemotherapy. The project is funded by the Ministry of Education's Humanities and Social Sciences Research Planning Fund in 2023. As you meet the criteria for participation, we invite you to join this study. The researchers will fully explain the contents of this informed consent form. Please read it carefully and make a thoughtful decision about whether to participate. If you are currently enrolled in another study, please inform the research team.

Purpose of the Study:

Gastric cancer is the third most prevalent cancer in China, and chemotherapy is a major adjuvant treatment post-surgery, which may cause psychological and symptomatic distress. Psychological resilience is the process through which individuals, when faced with internal and external pressures, activate their inherent cognitive abilities, competencies, or psychological traits, utilizing internal and external resources to repair and adjust mechanisms. This study, in line with the "Healthy China 2023 Action Plan," is based on the social-ecological system theory and seeks to explore strategies for enhancing the psychological resilience of cancer survivors through coordinated management by multiple stakeholders, ultimately promoting physical and mental health and improving well-being.

Our research team has previously developed and preliminarily tested a patient-centered, family-involved psychological resilience intervention program based on protective factors. The results indicated good feasibility, acceptability, and effectiveness. We are now conducting a large-scale clinical trial to further validate and refine the intervention program. Additionally, semi-structured interviews will be conducted to explore strategies for enhancing psychological resilience and to summarize the best evidence.

Study Procedures and Methods:

A convenience sampling method will be used to select gastric cancer patients undergoing chemotherapy at a tertiary hospital's oncology department between July 1, 2023, and June 30, 2026. Participants will be divided into a control group and an intervention group based on the order of admission. The control group will receive routine psychological care during chemotherapy, while the intervention group will receive an additional psychological resilience intervention program developed by our research team. The effectiveness of the intervention will be evaluated using outcome measures such as psychological resilience, hope levels, negative emotions (anxiety, depression), family functioning, social functioning, and biochemical indicators.

Process evaluation will be conducted alongside the intervention study, using semi-structured interviews to understand the mechanisms influencing the intervention. The combination of quantitative and qualitative methods will provide evidence for developing comprehensive strategies to enhance the psychological resilience of cancer survivors.

Potential Benefits:

This study aims to improve patients' psychological resilience and overall quality of life. Successful implementation of this study could provide valuable insights for

developing psychological resilience intervention programs for other chronic disease patients.

Risks and Discomforts:

The psychological resilience intervention provided to eligible participants in this study is non-invasive. Participants can withdraw from the study at any time during the intervention. All researchers involved have received standardized training.

Alternative Treatment Options:

Participants will not receive any alternative interventions or treatments other than those provided in this study.

Privacy and Confidentiality:

If you decide to participate in this study, your personal data collected during the study will be kept confidential.

Costs and Compensation:

All costs associated with the study, including technical and material expenses (e.g., printing fees), will be covered by the project funds. The study will not impose any additional financial burden on participants. During the study, some small gifts will be provided as rewards using project funds. Both patients and their families will directly benefit from this study.

Voluntary Participation and Right to Withdraw:

As a participant, you have the right to access information related to this study and its progress at any time. You may choose to participate or withdraw from the study at your discretion. Should you choose to withdraw, your medical treatment and rights will not be affected. If continuing in the study poses significant harm, the researchers will also terminate your participation. During the study, please provide truthful information regarding your medical history and current condition, report any discomfort experienced during the study, and inform the researchers if you are currently or have recently participated in other studies.

Contact Information:

If you have any questions related to this study, experience any discomfort or injury during the study, or have concerns about your rights as a participant, you can contact the study lead, Zhao Xia, at 1984017*.

Post-Trial Benefits:

The research findings will be shared through academic publications and conference presentations, providing valuable insights for the development of rehabilitation interventions for other cancer or chronic disease patients.

Consent to Participate:

I have read this informed consent form, and the research staff member (signature) has fully explained the purpose, content, risks, and benefits of this study to me. I have received answers to all my questions. I understand the study and voluntarily agree to participate.

Participant's Signature: ___________________________

Date: ________Year ________Month ________Day

(If the participant is illiterate, a witness's signature is required. If the participant is

incapacitated, a proxy's signature is required.)
