# Supplementary material for: Complex intervention based on protective factors to improve resilience for gastric cancer patients: Mixed-methods process evaluation protocol
Source: PLoS One. 2025 Aug 13;20(8):e0329834. doi: 10.1371/journal.pone.0329834 (PMC12349701; doi:10.1371/journal.pone.0329834)
Supplement: S6 File — (PDF) [file pone.0329834.s006.pdf]

Ethics committee approval letter（Chinese）：

编 号： 84230068

安徽医科大学生物医学伦理委员会
课题论证报告
（正　本）

课题负责人：章新琼

课题名称：多维协同视域下癌症幸存者心理弹性干预及其提升策略研究

承担单位：安徽医科大学

一、伦理委员会对该课题方案进行了论证，并特别对以下三方面进行了认真讨论：

1、研究对象的权利与利益；

2、确保取得知情同意的措施；

3、存在的危险与可能的受益。

二、同意实施该课题方案。实施过程中请使用经论证的知情同意书、问卷、说明信等材料。

三、课题方案如需修改，须事先经伦理委员会论证方可实施，修改内容及其原因需详细备案。

四、实施过程中如出现任何不良反应需立即向伦理委员会做出书面报告。

生效日期：2023年 7 月 11 日

安徽医科大学生物医学伦理委员会

（盖 章）

安徽医科大学生物医学伦理委员会

Ethics committee approval letter（English）：

Anhui Medical University Biomedical Ethics Committee
Ethics Review Report (Official)

Principal Investigator: Xinqiong Zhang

Study Title: Exploratory Research on Enhancing Psychological Resilience of Cancer Survivors through Multi-Community Collaboration

Affiliated Institution: Anhui Medical University

Ethics number: 84230068

The ethics committee has thoroughly reviewed the proposed study and specifically discussed the following three aspects:
1.The rights and welfare of the research participants;
2.Measures to ensure obtaining informed consent from participants;
3.Any potential risks and benefits to participants;

Approval is granted to conduct the proposed study. During implementation, please ensure the use of the approved consent forms, questionnaires, and explanatory documents.

Any modifications to the study must be submitted for ethical review and approval before implementation. Detailed documentation on the changes and reasons must be provided.

If any adverse events occur during the study, the ethics committee must be notified promptly.

Effective Date: July 11, 2023

Anhui Medical University Biomedical Ethics Committee

Special Explanation：

1.Ethical approval for the overall intervention study, including this process evaluation, was obtained from Anhui Medical University Biomedical Ethics Committee under approval number 84230068. The ethical review covers all relevant procedures related to the process evaluation, including participant consent and data collection methods.

2.In the overall intervention study, the research uses gastric cancer patients as an example for the intervention, but the ultimate goal is to develop a psychological resilience intervention program that can benefit all cancer patients. Therefore, the ethical approval was granted for a study population of cancer patients in general, while this process evaluation focuses on gastric cancer patients. As such, there is no conflict between the two.
