# Supplementary material for: Complex intervention based on protective factors to improve resilience for gastric cancer patients: Mixed-methods process evaluation protocol
Source: PLoS One. 2025 Aug 13;20(8):e0329834. doi: 10.1371/journal.pone.0329834 (PMC12349701; doi:10.1371/journal.pone.0329834)
Supplement: S7 File — (PDF) [file pone.0329834.s007.pdf]

Fund confirmation:

1.The Humanities and Social Sciences Research Planning Fund of Ministry of Education of China. （Chinese）：

教 育 部 司 局 函 件

教社科司函〔2017〕147 号

2017 年度教育部人文社会科学研究一般项目立项通知书

安徽医科大学 章新琼同志：

您申报的《癌症患者心理弹性预测因素研究：以保护性因素为中心》课题，经我部组织专家评审并经公示，正式批准为 2017 年度教育部人文社会科学研究规划基金项目。

项目批准号：17YJAZH126

批准经费：8 万元。项目经费实行规范的预决算管理，分年度进行拨款。该项目 2017 年度项目经费 4 万元，将由我部财务司于近期拨至你单位计划内财务账号。请按照《高等学校哲学社会科学繁荣计划专项资金管理办法》，严格执行国家有关科研经费管理制度，合理合规使用经费，提高经费使用效益。

立项时间：2017 年 7 月 27 日。项目研究周期一般为 3 年。我部将于 2019 年统一组织项目中期检查，年度预算执行情况是项目中期检查的内容之一。中期检查情况将作为后续拨款的重要依据。

请按照《教育部人文社会科学研究项目管理办法》的要求和您申报的《教育部人文社会科学研究项目申请评审书》中设计的研究内容及研究计划开展项目研究，确保项目按期保质保量完成。项目鉴定、结项按照《教育部人文社会科学研究项目成果鉴定和结项办法》（教社科司函[2007]145 号）进行。有关项目管理办法请登录中国高校人文社会科学信息网（www.sinoss.net）查询。所有出版或发表的项目研究成果，须在显著位置标明“教育部人文社会科学研究规划基金/青年基金/自筹经费项目”字样和项目批准号，否则项目中期检查及鉴定结项不予通过。

教育部社会科学司

2017年7月27日

The Humanities and Social Sciences Research Planning Fund of Ministry of Education of China. （English）：

Letter from the Department of Social Sciences of the Ministry of Education
Document No. (2017) 147 from the Department of Social Sciences

Notice of Project Approval for the 2017 General Project of the Ministry of Education's Humanities and Social Sciences Research Program

Dear Zhang Xinqiong from Anhui Medical University,

Your project titled "Research on Predictive Factors of Psychological Resilience in Cancer Patients: Focusing on Protective Factors" has been formally approved as a 2017 General Project of the Ministry of Education's Humanities and Social Sciences Research Program, following expert review and public notification.

Project Approval Number: 17YJAZH126
Approved Funding: 80,000 RMB. The project funds will be managed under a regulated pre- and post-budget management system and disbursed annually. For the 2017 fiscal year, a sum of 40,000 RMB will be transferred to your institution's designated financial account by the Ministry's Finance Department in the near future. Please ensure compliance with the "Regulations on the Management of Special Funds for the Prosperity of Philosophy and Social Sciences in Higher Education Institutions" and strictly adhere to national regulations on research fund management. The funds should be used appropriately and efficiently.

Project Approval Date: July 27, 2017. The project’s research period is generally three years. Our department will organize a mid-term review of the project in 2019, and the project's budget execution for each fiscal year will be part of the mid-term review. The results of the mid-term review will serve as a key basis for subsequent funding allocations.

Please conduct your research in accordance with the requirements outlined in the "Regulations on the Management of Humanities and Social Sciences Research Projects of the Ministry of Education" and the research content and plan submitted in your project proposal for the Ministry of Education's Humanities and Social Sciences Research Program. Ensure that the project is completed on time and with high quality. Project evaluations and closures will be conducted according to the "Measures for the Evaluation and Closure of Achievements of the Ministry of Education's Humanities and Social Sciences Research Projects" (Document No. 145 [2007] from the Department of Social Sciences). Relevant management regulations can be found on the website of the China Higher Education Humanities and Social Sciences

Information Network (www.sinoss.net). All published or released research outcomes from the project must prominently indicate "Supported by the Ministry of Education's Humanities and Social Sciences Research Program/Youth Fund/Self-funded Project" along with the project approval number. Projects that fail the mid-term review or final evaluation will not receive subsequent funding.

Department of Social Sciences, Ministry of Education
July 27, 2017

2.Fund of the Anhui Provincial Department of Education. （Chinese）:

安 徽 省 教 育 厅

皖教秘科〔2021〕104 号

安徽省教育厅关于 2021 年度高校科学研究项目立项的通知

各高等学校：

根据《安徽省教育厅关于开展 2021 年度高校科学研究项目申报工作的通知》（皖教秘科〔2021〕63 号），经学校组织申报、评审、公示无异议和省教育厅审核批准，现将 2021 年度高校科学研究项目予以下达（见附件 1、2、3）。请各高校认真对照《安徽省教育厅科学研究项目管理办法》（皖教科〔2017〕2 号）相关要求组织实施。

一、切实加强项目管理

各高校要加强对立项科研项目的管理，督促项目承担人精心组织项目实施，按时完成项目研究任务，提高项目完成质量，为争取国家级和省部级科研项目奠定基础，为提升学科建设水平提供支撑。同时，加强对科研项目的结题管理与评价，进一步强化质量和绩效导向，加强分类绩效评价，强化绩效评价结果运用。省教育厅下一步将对各高校科学研究项目结题管理和评价情况进行抽查，将项目绩效评价结果作为后续项目支持的重要依据。

二、严格落实项目经费

各高校要严格按照皖教秘科〔2021〕63 号文件要求落实项目

支持经费，并积极贯彻落实国家和安徽省有关科研经费管理的政策文件，扩大科研项目经费管理自主权，减轻科研人员事务性负担，加大科研人员激励力度，修订完善学校相关项目资金管理办法。省教育厅将对各高校落实项目经费和管理责任情况进行抽查，对未按承诺落实的高校将核减其下一年度项目立项指标。

三、强化科研育人功能

各高校要引导师生树立正确的政治方向、价值取向、学术导向，鼓励科研人员将科研创新资源转化为高质量育人资源，将科研创新优势转化为育人优势，鼓励、支持在校学生参与项目研究，支持有研究能力的学生早进实验室、早进团队、早进项目，培养大学生的创新意识和能力，营造科研育人浓厚氛围。

附件：1.2021 年度安徽高校自然科学研究项目一览表

2.2021 年度安徽高校人文社会科学研究项目一览表

3.2021 年度安徽高校研究生科学研究项目一览表

安徽省教育厅

2021 年 12 月 24 日

（此件依申请公开）

附件3

2021年度安徽高校研究生科学研究项目一览表

42	安徽医科大学	基于保护性因素的化疗期胃癌患者心理弹性干预方案的构建及初步应用	丁雅楠	一般项目	YJS20210299

Fund of the Anhui Provincial Department of Education.（English）:

Anhui Provincial Department of Education
Secretariat of Science Document No. [2021] 104
Notice on the Approval of University Scientific Research Projects for the Year 2021

To all higher education institutions:
Following the "Notice on the Implementation of the 2021 University Scientific Research Project Application Work" (Secretariat of Science Document No. [2021] 63), and after organized application, review, public notification without objections, and approval by the Provincial Department of Education, the 2021 university scientific research projects are hereby approved (see Attachments 1, 2, and 3). All universities are required to implement these projects carefully in accordance with the "Anhui Provincial Department of Education's Management Methods for Scientific Research Projects" (Science Document No. [2017] 2).
I. Strengthen Project Management
Universities must enhance the management of approved scientific research projects, ensuring project leaders organize and implement the projects meticulously, complete the research tasks on time, and enhance the quality of outcomes. This will lay the foundation for securing national and provincial-level research projects and support the enhancement of academic construction levels. Moreover, strengthen the management and evaluation of project completion to further emphasize quality and performance orientation, enhance categorized performance evaluations, and utilize the results of performance evaluations. The Provincial Department of Education will conduct spot checks on the management and evaluation of scientific research projects at universities, using the results of performance evaluations as a critical basis for future project funding.
II. Strictly Implement Project Funding
Universities must strictly adhere to the stipulations in Document No. [2021] 63 for implementing project funding, actively apply national and Anhui provincial policies on the management of scientific research funds, expand the autonomy in managing project funds, reduce the administrative burden on researchers, and increase incentives for researchers. The Provincial Department of Education will spot-check the implementation of project funding and management responsibilities at universities, and schools that do not fulfill their commitments will have their project approval quotas for the next year reduced.
III. Enhance the Educational Role of Scientific Research
Universities should guide teachers and students to establish correct political, value, and academic orientations. Encourage researchers to transform scientific innovation resources into high-quality educational resources and research advantages into educational benefits. Encourage and support students to participate in research projects, promoting early involvement in laboratories, teams, and projects to cultivate

university students' innovative consciousness and capabilities, and create a robust environment for educational engagement through research.

Attachments:

1.List of Natural Science Research Projects for Anhui Universities in 2021.

2.List of Humanities and Social Sciences Research Projects for Anhui Universities in 2021.

3.List of Postgraduate Scientific Research Projects for Anhui Universities in 2021.

Attachment 3: List of Postgraduate Scientific Research Projects for Anhui Universities in 2021.

42.

Anhui Medical University

Development and Preliminary Application of a Psychological Resilience Intervention Program Based on Protective Factors for Gastric Cancer Patients Undergoing Chemotherapy

Principal Investigator: Ding Yanan

General Project

Project Number: YJS20210299
