# Supplementary material for: Complex intervention based on protective factors to improve resilience for gastric cancer patients: Mixed-methods process evaluation protocol
Source: PLoS One. 2025 Aug 13;20(8):e0329834. doi: 10.1371/journal.pone.0329834 (PMC12349701; doi:10.1371/journal.pone.0329834)
Supplement: Attachment — Submitted filename: PONE-D-24-38986_reviewer with suggestions.pdf [file pone.0329834.s008.pdf]

PLOS ONE

Complex intervention based on protective factors to improve resilience for gastric cancer patients: mixed-methods process evaluation protocol

--Manuscript Draft--

**Manuscript Number:**	PONE-D-24-38986
**Article Type:**	Study Protocol
**Full Title:**	Complex intervention based on protective factors to improve resilience for gastric cancer patients: mixed-methods process evaluation protocol
**Short Title:**	Short Title - Improving resilience in gastric cancer: mixed-methods process evaluation protocol
**Corresponding Author:**	Zhang Xinqiong<br>Anhui Medical University<br>CHINA
**Keywords:**	
**Abstract:**	Abstract<br>Background<br>Gastric cancer represents a global health burden, with patients often experiencing significant psychological distress due to various treatments. Resilience, the ability to adapt positively to challenges, is crucial for the mental and physical well-being of gastric cancer patients. Despite its importance, there are still gaps in research aimed at enhancing resilience. This paper presents a process evaluation protocol embedded within a complex intervention aimed at enhancing resilience in gastric cancer patients during chemotherapy. The protocol aims to understand the implementation process, mechanisms, influencing factors, and outcomes.<br>Methods<br>The process evaluation will use a mixed-methods approach. The Consolidated Framework for Implementation Research (CFIR) and the RE-AIM<br>framework were chosen to qualitatively identify the factors that influence intervention implementation and the outcomes of implementation, including the reach, efficacy, adoption and maintenance. The implementation process will be assessed based on the UK Medical Research Council's guidance on evaluating complex interventions. The fidelity, dose and adherence will be analysed through the goal achievement log book, and the mechanisms of intervention implementation will be assessed using parallel latent growth curve model with quantitative information. We will conduct semi-structured interviews with intervention staff and participants, analyse data from the goal achievement log book, and analyse data from the primary and secondary outcome indicators for parallel latent growth curve parameters.<br>Discussion<br>This study outlines a process evaluation protocol for a complex intervention. The protocol aims to further validate the effectiveness of the intervention and guide the development of more comprehensive resilience strategies, thus promoting broader application among other chronic disease patient populations.<br>Trial registration<br>The trial has been prospectively registered on 11 July 2023; ChiCTR2300073466.<br>Keywords Gastrointestinal tumours, Protocols & guidelines, Mental health, Psychosocial intervention, Qualitative research
**Order of Authors:**	Xia Zhao
	Min Fan
	Yanan Ding
	Yuqin Pan
	Zhang Xinqiong
**Additional Information:**	
**Question**	**Response**

Powered by Editorial Manager® and ProduXion Manager® from Aries Systems Corporation

**Financial Disclosure**<br><br>Enter a financial disclosure statement that describes the sources of funding for the work included in this submission. Review the submission guidelines for detailed requirements. View published research articles from *PLOS ONE* for specific examples.<br><br>This statement is required for submission and **will appear in the published article** if the submission is accepted. Please make sure it is accurate.<br><br>**Funded studies**<br>Enter a statement with the following details:<br>• Initials of the authors who received each award<br>• Grant numbers awarded to each author<br>• The full name of each funder<br>• URL of each funder website<br>• Did the sponsors or funders play any role in the study design, data collection and analysis, decision to publish, or preparation of the manuscript?<br><br>Did you receive funding for this work?	Yes
Please add funding details.<br>as follow-up to "**Financial Disclosure**<br><br>Enter a financial disclosure statement that describes the sources of funding for the work included in this submission. Review the submission guidelines for detailed requirements. View published research articles from *PLOS ONE* for specific examples.<br><br>This statement is required for submission and **will appear in the published article** if the submission is accepted. Please make sure it is accurate.	This paper was supported by the Humanities and Social Sciences Research Planning Fund of Ministry of Education of China (grant number: 17YJAZH126) and the Anhui Provincial Department of Education (grant number: yjs20210299).

**Funded studies**<br>Enter a statement with the following details:<br>• Initials of the authors who received each award<br>• Grant numbers awarded to each author<br>• The full name of each funder<br>• URL of each funder website<br>• Did the sponsors or funders play any role in the study design, data collection and analysis, decision to publish, or preparation of the manuscript?<br><br>Did you receive funding for this work?"	
Please select the country of your main research funder (please select carefully as in some cases this is used in fee calculation).<br>as follow-up to "**Financial Disclosure**<br><br>Enter a financial disclosure statement that describes the sources of funding for the work included in this submission. Review the submission guidelines for detailed requirements. View published research articles from *PLOS ONE* for specific examples.<br><br>This statement is required for submission and **will appear in the published article** if the submission is accepted. Please make sure it is accurate.<br><br>**Funded studies**<br>Enter a statement with the following details:<br>• Initials of the authors who received each award<br>• Grant numbers awarded to each author<br>• The full name of each funder<br>• URL of each funder website<br>• Did the sponsors or funders play any role in the study design, data collection and analysis, decision to publish, or preparation of the manuscript?<br><br>Did you receive funding for this work?"	CHINA - CN
**Competing Interests**	The authors have declared that no competing interests exist.

Use the instructions below to enter a competing interest statement for this submission. On behalf of all authors, disclose any competing interests that could be perceived to bias this work—acknowledging all financial support and any other relevant financial or non-financial competing interests.<br><br>This statement is required for submission and **will appear in the published article** if the submission is accepted. Please make sure it is accurate and that any funding sources listed in your Funding Information later in the submission form are also declared in your Financial Disclosure statement.<br><br>View published research articles from *PLOS ONE* for specific examples.<br><br>**NO authors have competing interests**<br><br>Enter: *The authors have declared that no competing interests exist.*<br><br>**Authors with competing interests**<br><br>Enter competing interest details beginning with this statement:<br><br>*I have read the journal's policy and the authors of this manuscript have the following competing interests: [insert competing interests here]*<br><br>* typeset	
**Ethics Statement**<br><br>Enter an ethics statement for this submission. This statement is required if the study involved:<br><br>• Human participants<br>• Human specimens or tissue<br>• Vertebrate animals or cephalopods<br>• Vertebrate embryos or tissues	This study has received ethical approval on July 11th, 2023, from the Ethics Committee of Anhui Medical University, China (ethics number: 84230068).

Powered by Editorial Manager® and ProduXion Manager® from Aries Systems Corporation

• Field research<br><br>Write "N/A" if the submission does not require an ethics statement.<br><br>General guidance is provided below. Consult the submission guidelines for detailed instructions. **Make sure that all information entered here is included in the Methods section of the manuscript.**	

Powered by Editorial Manager® and ProduXion Manager® from Aries Systems Corporation

**Format for specific study types**<br><br>**Human Subject Research (involving human participants and/or tissue)**<br>• Give the name of the institutional review board or ethics committee that approved the study<br>• Include the approval number and/or a statement indicating approval of this research<br>• Indicate the form of consent obtained (written/oral) or the reason that consent was not obtained (e.g. the data were analyzed anonymously)<br><br>**Animal Research (involving vertebrate animals, embryos or tissues)**<br>• Provide the name of the Institutional Animal Care and Use Committee (IACUC) or other relevant ethics board that reviewed the study protocol, and indicate whether they approved this research or granted a formal waiver of ethical approval<br>• Include an approval number if one was obtained<br>• If the study involved *non-human primates*, add *additional details* about animal welfare and steps taken to ameliorate suffering<br>• If anesthesia, euthanasia, or any kind of animal sacrifice is part of the study, include briefly which substances and/or methods were applied<br><br>**Field Research**<br><br>Include the following details if this study involves the collection of plant, animal, or other materials from a natural setting:<br>• Field permit number<br>• Name of the institution or relevant body that granted permission	
**Data Availability**<br><br>Authors are required to make the data underlying their research findings fully available, without restriction.<br><br>If the manuscript reports pilot or preliminary results, the data underlying those results must be made available at the time of publication. A Data Availability	Yes - Results are reported and the data are fully available

Statement describing where the data can be found is required at submission. Your answers to this question constitute the Data Availability Statement and will be published in the article, if accepted. When legal or ethical requirements prohibit public data sharing, authors must indicate how researchers may obtain access to the data. See the PLOS Data Policy and FAQ for detailed information.<br><br>**Important:** Stating 'data available on request from the author' is not sufficient. If your data are only available upon request, select 'No' for the first question and explain your situation in the text box.<br><br>For protocols without pilot or preliminary data, authors are strongly encouraged to state how they plan to share research data from their study when it is completed or published.<br><br>If your study includes pilot data, will they be made fully available without restriction?	
**Describe where the data may be found in full sentences. If you are copying our sample text, replace any instances of XXX with the appropriate details.**<br><br>• If **no pilot data are reported** in the manuscript, please enter the following: *No datasets were generated or analysed during the current study. All relevant data from this study will be made available upon study completion.*<br>• If the manuscript reports pilot data **held in a public repository**, include URLs, accession numbers or DOIs. If this information will only be available after acceptance, indicate this by ticking the box below. For example: *All XXX files are available from the XXX database (accession number(s) XXX, XXX.).*<br>• If the manuscript reports pilot data contained **within the manuscript and/or**	No datasets were generated or analysed during the current study. All relevant data from this study will be made available upon study completion.

**Supporting Information files**, enter the following:<br>*All relevant data are within the manuscript and its Supporting Information files.*<br>• If neither of these applies but you are able to provide **details of access elsewhere**, with or without limitations, please do so. For example:<br><br>*Data cannot be shared publicly because of [XXX]. Data are available from the XXX Institutional Data Access / Ethics Committee (contact via XXX) for researchers who meet the criteria for access to confidential data.*<br><br>*The data underlying the results presented in the study are available from (include the name of the third party and contact information or URL).*<br>• This text is appropriate if the data are owned by a third party and authors do not have permission to share the data.<br><br>For study protocols with data management or sharing plans, authors are encouraged to briefly describe in their Data Availability Statement how data they generate will be made accessible when the study is completed. For example:<br>• Deidentified research data will be made publicly available when the study is completed and published.<br><br>* typeset	
Additional data availability information:	

Powered by Editorial Manager® and ProduXion Manager® from Aries Systems Corporation

Manuscript Click here to access/download;Manuscript;Manuscript.docx

Complex intervention based on protective factors to improve resilience for gastric cancer patients: mixed-methods process evaluation protocol

Xia Zhao[1], Min Fan[1], Yanan Ding [2],Yuqin Pan [3],Xinqiong Zhang[1*]

[1]School of Nursing, Anhui Medical University, Hefei, Anhui, China.
[2]Anhui Sanlian University, Hefei, Anhui, China.
[3]Department of Oncology, The First Affiliated Hospital of Anhui Medical University, Hefei, Anhui, China.

[*]Xinqiong Zhang: Corresponding author; E-mail: hixqzhang@163.com(XZ)

Abstract

Background

Gastric cancer represents a global health burden, with patients often experiencing significant psychological distress due to various treatments. Resilience, the ability to adapt positively to challenges, is crucial for the mental and physical well-being of gastric cancer patients. Despite its importance, there are still gaps in research aimed at enhancing resilience. This paper presents a process evaluation protocol embedded within a complex intervention aimed at enhancing resilience in gastric cancer patients during chemotherapy. The protocol aims to understand the implementation process, mechanisms, influencing factors, and outcomes.

Methods

The process evaluation will use a mixed-methods approach. The Consolidated Framework for Implementation Research (CFIR) and the RE-AIM framework were chosen to qualitatively identify the factors that influence intervention implementation

and the outcomes of implementation, including the reach, efficacy, adoption and maintenance. The implementation process will be assessed based on the UK Medical Research Council's guidance on evaluating complex interventions. The fidelity, dose and adherence will be analysed through the goal achievement log book, and the mechanisms of intervention implementation will be assessed using parallel latent growth curve model with quantitative information. We will conduct semi-structured interviews with intervention staff and participants, analyse data from the goal achievement log book, and analyse data from the primary and secondary outcome indicators for parallel latent growth curve parameters.

Discussion

This study outlines a process evaluation protocol for a complex intervention. The protocol aims to further validate the effectiveness of the intervention and guide the development of more comprehensive resilience strategies, thus promoting broader application among other chronic disease patient populations.

Trial registration

The trial has been prospectively registered on 11 July 2023; ChiCTR2300073466.

Keywords Gastrointestinal tumours, Protocols & guidelines, Mental health, Psychosocial intervention, Qualitative research

Introduction

According to the latest statistics from the International Agency for Research on Cancer (IARC)[1], gastric cancer (GC) ranked fifth globally in terms of both new

cases and deaths in 2022, highlighting its significant burden on global public health. As a fatal disease[2-3], GC patients face various treatment modalities[4], including surgery and chemotherapy, and suffer severe psychological distress such as depression, anxiety, and psychological challenges. Research[5] has shown that patients who adopt positive coping strategies are more likely to regulate, adapt, and grow with a positive and optimistic mindset when facing physical and psychological challenges. With the development of positive psychology, the study of resilience, focusing on individuals' positive psychological responses, has become a research hotspot[6-7]. Studies have demonstrated[8] a close correlation between resilience in GC patients and their likelihood of survival; higher resilience is associated with greater confidence in facing the disease. Numerous studies have explored interventions that promote psychological resilience. Research[9] has showed that psychological health interventions targeting resilience can effectively improve symptoms and reduce suffering in cancer patients. However, existing interventions mostly rely on general psychological therapies such as attention and interpretation therapy and cognitive interventions, lacking targeted interventions focusing on resilience itself. In a large-sample longitudinal study spanning six months[10], our research team investigated psychological resilience among chemotherapy-treated GC patients, identifying social function, family function, self-efficacy, and hope as protective factors contributing to resilience in these patients. Based on these findings, our team developed an intervention program centered around protective factors of psychological resilience aimed at enhancing resilience during chemotherapy for GC patients. A pilot study demonstrated[11] feasibility and

acceptability of this intervention. To further validate its effectiveness and refine intervention strategies, we are conducting a large-sample formal trial study, currently underway.

The large-sample chemotherapy-phase GC patient resilience intervention study (referred to as "resilience intervention" below) is a single-blind, quasi-experimental study. Due to ethical and practical considerations, we opted for a non-concurrent quasi-experimental design. Given the nature of the intervention, blinding of researchers and patients was not feasible; however, single-blinding was implemented for outcome assessors. Before implementing the intervention, the research team members and clinical nurses, all of whom hold psychological counseling certification, were trained by the intervention developers to provide detailed instructions on the intervention and its procedures.

Convenience sampling was used to select 196 chemotherapy-phase GC patients treated at a tertiary hospital in China as study participants, all meeting inclusion and exclusion criteria (detailed criteria as per the pilot study[11]). Following patient informed consent, those admitted from August 2023 to March 2024 were assigned to the control group, while those admitted from April 2024 to December 2024 were assigned to the intervention group. Baseline assessments were conducted to collect primary outcome measures, specifically resilience levels, and secondary outcome measures, including levels of hope, negative emotions (anxiety, depression), family function, social function, C-reactive protein, cortisol, and interleukin. Resilience levels, hope levels, negative emotional levels (anxiety, depression), family function,

and social function will be assessed respectively through the 14-Item Resilience Scale[12], Herth Hope Index[13], Self-Rating Anxiety Scale[14], Self-Rating Depression Scale[15], the Chinese version of the Family Hardiness Index (C-FHI)[16], and the Social Support Rating Scale[17] for data collection. C-reactive protein, cortisol, and interleukins will be obtained through the hospital database.

After baseline data collection, the control group received standard psychological care measures (see Table 1). The intervention group underwent the resilience intervention, administered by the research team and clinical nurses. The intervention spanned two months with six sessions, each lasting approximately 30-50 minutes. A blended approach combining online and in-person methods was utilized: researchers conducted face-to-face sessions with patients during hospitalization, supplemented by online sessions during chemotherapy intervals. Each session included a video segment accessible via QR code for patient viewing or facilitated by intervention staff. These videos aimed to enhance patients' understanding of the intervention process and content. Table 2 provides an overview of the intervention content; detailed intervention information can be found in the pilot study[11]. Following the intervention period, outcome measures were again assessed in both groups to evaluate the intervention's effectiveness based on changes in primary and secondary outcome indicators.

Table 1. Control group psychological nursing content.

Care measure	Care content
Nursing assessment	Assess psychological, familial, and social support, mental status, and disease perception.
Nursing interventions	①Condition monitoring: Closely monitor the patient's psychological changes, communicate promptly with the patient's family, and prevent adverse events; ②Symptomatic nursing: Provide targeted guidance to the patient to stabilize emotions; ③Medication care: Implement nursing interventions tailored to different medications to alleviate the patient's anxiety and tension.
Health education	①At Admission: Engage in proactive communication with the patient, introduce the responsible physician and nursing staff, and facilitate the patient's adaptation to the ward environment; ②During Hospitalization: Maintain a positive attitude, actively engage in treatment, clarify patient doubts, and offer support as needed; ③At Discharge: Provide guidance on self-management of chemotherapy side effects and emphasize the importance of regular follow-up appointments and monitoring of relevant health indicators.

Table 2. Overview of the resilience intervention.

Intervention sessions	Intervention themes	Intervention content	Intervention form	Intervention staff
No.1	Fostering trust	1. Establish a good relationship<br>2. Introduce the research objectives<br>3. Show videos and distribute the goal achievement log book	Face-to-face	Team members & clinical nurses
No.2	Goal setting	Guide the setting of appropriate goals	Face-to-face	Team members & clinical nurses
No.3	Family function	1. Guide review of family support<br>2. Watch intervention videos<br>3. Assign homework	WeChat	Team members
No.4	Self-efficacy	1. Role model inspiration<br>2. Encourage positive thinking<br>3. Assign homework	Face-to-face	Team members & clinical nurses
No.5	Social support	1. Provide social and peer support<br>2. Assign homework	WeChat	Team members
No.6	Self-efficacy strengthening	1. Guide reverse thinking<br>2. Show videos on emotional regulation<br>3. Assign homework	Face-to-face	Team members & clinical nurses

Complex interventions share two common features: the complexity of the intervention itself and the complexity of its pathways. Process evaluation[18] is a crucial component in designing and testing complex interventions, equally important as outcome evaluation. [19]The resilience intervention comprises several interacting components and diverse intervention measures, contributing to its pathway complexity. Process evaluation is necessary to identify the mechanisms of each intervention component and the underlying logic behind the outcomes. Therefore, this study elaborates on the process evaluation protocol for the resilience intervention. The aim of the protocol is to understand the implementation process and influencing factors, complementing the outcome evaluation by illustrating the impact of process evaluation on the results, thereby providing insights for developing more refined intervention strategies and broader application.

Specific objectives are to:

1. Assessing the reach, efficacy, adoption, maintenance, fidelity, dose, and adherence of the intervention implementation.

2. Exploring the mechanisms of the intervention measures and the impact of process on intervention results.

3. Exploring the facilitating and barrier factors of intervention implementation from the stakeholders' perspective, as well as their views on the intervention.

Materials and Methods

Design

This study employs a mixed-methods approach integrating qualitative and quantitative data, focusing on the process, mechanisms, factors, and outcomes of intervention under effectiveness trial context.

Process evaluation[18] follows the guidance of the Medical Research Council (MRC) framework. This framework directs several stages of complex intervention: development of intervention measures, feasibility assessment in the pilot phase, the implementation, and evaluation in the formal trial stage. During the formal trial stage, MRC emphasizes assessing the quantity and quality of intervention delivery content to provide greater confidence in effectiveness conclusions. At the beginning of the intervention, the intervention staff will distribute goal achievement log books (S1 File) to the patients in the intervention group to record fidelity, dose, and adherence of implementation processes. We will quantitatively assess the delivery of the intervention through data analysis after its completion.

For intervention mechanisms, we will employ Parallel Latent Growth Curves[20] with quantitative data to explain how the intervention produces effects and the underlying processes of these effects. [21]Parallel Latent Growth Curves is commonly used in psychology, social sciences, medicine, and other fields to explore potential trends in variable changes over time and whether differences exist between individuals or groups in these changes. In intervention research, Parallel Latent

Growth Curves can assess the intervention's impact on changes in outcome variables over time.

Additionally, we will use the Consolidated Framework for Implementation Research (CFIR) and the RE-AIM framework to guide the determination of influencing factors and outcomes. CFIR[22] is a meta-theoretical framework for implementation that comprehensively categorizes specific constructs related to intervention. These include intervention characteristics, inner and outer setting, characteristics of individuals, and process. CFIR will be used to guide the exploration of factors influencing the implementation process.The RE-AIM framework[23] will further guide our evaluation of the implementation outcomes, including (1) Reach: the proportion of the target population that participates in the intervention, (2) Effectiveness: the impact of the intervention, (3) Adoption: the willingness of the target population to adopt the intervention, (4) Implementation: the implementation of the intervention in real-world setting, (5) Maintenance: the sustainability of the intervention after its implementation. We will utilize semi-structured interviews to collect qualitative data for identifying influencing factors and outcomes.

The study description follows the SPIRIT checklist(S2 File) , with detailed enrollment, intervention, and assessment timelines provided in Fig 1.

Fig 1. SPIRIT schedule of enrolment, data collection and assessments.

Logic model

The Implementation Research Logic Model (IRLM)[24] is being employed to

design the process evaluation(Fig 2). The IRLM provides a method to describe the complex relationships between key elements of implementation research and practice, enhancing rigor and replicability in research and implementation practice. The IRLM specifies the following core elements of implementation research: (1) determinants of implementation, (2) implementation strategies, (3) mechanisms, (4) outcomes. The IRLM offers a systematic and comprehensive approach to integrate resources from various stakeholders. This model will be used to present and explain our findings and to test whether potential causal hypotheses are correct.

Fig 2. The logical model of process evaluation. GC, gastric cancer.

Data collection and analysis

[25]Limbani *et al* suggested that collecting process evaluation data before outcome data are available could aid in exploring implementation process without inadvertently influencing researcher or patient behaviors in the study. As this protocol is embedded within a large-sample formal trial, during the effectiveness research phase, to ensure the accuracy and reliability of study results, we will collect goal achievement log book and gather qualitative data through semi-structured interviews after the intervention has concluded. Quantitative data analysis will also be conducted. Details regarding outcome data collection methods and contents are outlined in Table 3.

Table 3. Process evaluation outcome data collection methods.

Outcome	Description	Method of evaluation
Reach	The proportion of the target population that participates in the intervention.	Semi-structured interview
Efficacy	Effect of intervention measures under ideal conditions.	Semi-structured interview
Adoption	Patient acceptance of the intervention.	Semi-structured interview
Maintenance	Possibility and necessity of sustained intervention use.	Semi-structured interview
Fidelity	The degree to which an intervention is delivered as intended.	Goal achievement log data analysis
Dose	Amount of intervention delivered to participants.	Goal achievement log data analysis
Adherence	The degree to which participants conform to the intervention protocol.	Goal achievement log data analysis
Parallel latent growth curve models for primary and secondary outcome data in intervention and control groups	Parallel latent growth curves of primary and secondary outcomes for intervention and control group patients over time, along with the differences between the curves.	Analyse intervention outcome data for parallel latent growth curve parameters

Qualitative data collection and analysis

Semi-structured interviews with intervention staff and participants:

Firstly, we will conduct semi-structured interviews with intervention staff to identify factors influencing intervention implementation and gather their perspectives on the intervention. The interview guide will be developed and refined using the question repository provided on the CFIR website (https://cfirguide.org/constructs/), which includes intervention characteristics (relative advantage, complexity, adaptability), inner setting (compatibility, access to knowledge and information, networks and communications), outer setting (external pressures, partnerships and

connections), characteristics of individuals (capability), processes (engaging, implementation, adapting, maintenance, reflecting and evaluating).

Next, we will conduct interviews with intervention participants with the aim to determine from their perspective: (1) factors influencing intervention implementation and their views on the intervention; (2) reach; (3) efficacy; (4) adoption; (5) maintenance. The interview guide development will be based on the theoretical approaches mentioned above. Determinants will be based on the CFIR framework, including intervention characteristics (relative advantage, complexity, adaptability), inner setting (compatibility, space, access to knowledge and information), outer setting (external pressures, partnerships and connections), characteristics of individuals (need, capability, motivation), and process (engaging, adapting, reflecting and evaluating). Reach, efficacy, adoption, and maintenance will be determined based on the RE-AIM framework.

Interview participants will be purposively sampled from the intervention group patients, following the principle of maximum variation sampling, selecting patients with significant differences in age, education level, occupation, religious beliefs, etc, until data saturation is reached, meaning no new themes emerge.

All interviews will be conducted in private, quiet hospital interview rooms after informing participants about the purpose and methods of the interview and obtaining their informed consent.The interviews will be conducted in person, one-on-one, by external staff who are independent of the intervention and experienced in qualitative data collection. Each interview will last 30 minutes or longer, recorded via audio

recording, anonymized, and transcribed verbatim. Data organization and analysis will be conducted using NVivo 11.0 software, and the Colaizzi phenomenological 7-step analysis method will be used to organize the interview recordings. Interview guides for intervention staff and participants are provided in S3 File.

Quantitative data collection and analysis

Parallel latent growth curve model analysis:

In addition to qualitative data, we will collect quantitative data with the aim of exploring the mechanisms of the intervention and the process of change, as well as the intervention's impact on outcomes. We will employ Parallel Latent Growth Curve model[20], which allow simultaneous analysis of multiple outcome variables and account for individual differences . The primary outcome (resilience level) and secondary outcomes (hope level, anxiety and depression levels, family function, social function, C-reactive protein, cortisol, interleukins) for both the intervention and control groups will be collected one day before the end of each chemotherapy hospitalization. These data will be collected three times, with each collection separated by one chemotherapy cycle. Data analysis will be conducted using R software. Model specifications will define growth factors for each outcome variable, assuming linear growth trajectories captured by time variables. Random effects will include random intercepts and slopes to allow for variability in growth trajectories between individuals. We will use Maximum Likelihood Estimation (MLE) to estimate model parameters and evaluate model fit using indices including Comparative Fit

Index (CFI), Tucker-Lewis Index (TLI), and Root Mean Square Error of Approximation (RMSEA). The significance level will be set at $p < 0.05$.

Goal achievement log book analysis:

After the final intervention session, we will collect goal achievement log book from patients to assess fidelity, dose, and adherence of implementation, aiming to understand the delivery of the intervention. Firstly, the text will undergo cleaning, which includes removing irrelevant content, formatting, and preparing the text for analysis. Secondly, we will carefully review the content of the logs: fidelity will be reflected by the percentage of intervention plan completed by participants, dose will be assessed by the duration of participant engagement in the intervention, and adherence will be evaluated by the percentage of intervention tasks completed by participants. Finally, descriptive statistics will be used to describe the central tendency and variability of fidelity, dose, and adherence. This process will be conducted by external staff independent of the intervention.

Ethics statement

This study has received ethical approval on July 11th, 2023, from the Ethics Committee of Anhui Medical University, China (ethics number: 84230068). All participants in the study signed an informed consent form after being informed of the study's purpose, and submitted it on paper. Participation in the process evaluation was entirely voluntary, and all participants had the right to refuse to participate and withdraw from the study at any time.

Discussion

Resilience intervention is a complex process based on protective factors, aimed at enhancing the resilience of GC patients during chemotherapy. To fully understand the intervention, it is essential not only to measure its effectiveness but also to explore the intricate processes involved, which will provide deeper insights into the internal mechanisms and interrelationships among the various components. This process evaluation protocol adheres to the MRC framework, CFIR framework, and RE-AIM framework to ensure scientific rigor and completeness. An embedded design, utilizing the IRLM, is employed to better understand the core implementation elements, including determinants, implementation strategies, mechanisms, outcomes, and potential causal relationships. A mixed-methods design is adopted, integrating qualitative and quantitative data to comprehensively reflect the implementation. The study also includes both intervention staff and participants, exploring influencing factors and perceptions of the intervention from multiple perspectives. The findings will provide valuable insights into improving resilience in GC patients and aid in the development of more personalized and effective psychosocial care strategies.

However, the study design has certain limitations. First, the research is conducted at a single tertiary hospital in China, which may introduce geographical bias and limit the generalizability of the findings. Second, participant bias may be present, as those who consented to and completed the intervention might be more inclined to seek help for their physical and psychological issues, and more likely to

adopt a positive attitude when facing challenges. Additionally, the single-blind design, where researchers and patients are not blinded, might lead to bias, although the outcome assessors were blinded to mitigate this effect.

To widely disseminate the research findings, we plan to submit the results of the process evaluation for publication in peer-reviewed journals and share them in scientific publications. We will also present our findings at academic conferences and seminars, engaging with healthcare professionals, psychologists, and nursing experts to promote the further development and application of the intervention.

Throughout the study, we may make necessary modifications to the intervention based on process evaluation results and participant feedback. These adjustments aim to enhance the effectiveness and acceptability of the intervention, ensuring it better meets the needs of patients. In extreme cases, if the intervention proves ineffective or harmful to participants, we will consider terminating the study and promptly report this to all relevant parties.

In conclusion, this process evaluation will provide critical empirical support for resilience interventions in GC patients and guide future research and practice. Through continuous evaluation and improvement, we aim to offer higher quality psychosocial support to GC patients and other chronic disease populations, helping them better cope with the challenges posed by their conditions.

Supporting Information

S1 Fig. SPIRIT schedule of enrolment, data collection and assessments.

S2 Fig. The logical model of process evaluation. GC, gastric cancer.

S1 File. Goal achievement log book. (DOCX)

S2 File. SPIRIT_Fillable-checklist. (DOC)

S3 File. Interview guides. (DOCX)

S4 File. Approved study protocol by ethics committee. (PDF)

Acknowledgements

We would like to express our gratitude to the professionals, patients, and their families who participated in this study.

Authors' contributions

Conceptualization: Xia Zhao, Min Fan, Yanan Ding, Yuqin Pan, Xinqiong Zhang.

Data curation: Xia Zhao, Min Fan, Yuqin Pan.

Formal analysis: Xia Zhao, Min Fan.

Funding acquisition: Yanan Ding, Xinqiong Zhang.

Investigation: Xia Zhao, Min Fan, Yuqin Pan.

Methodology: Xia Zhao, Yanan Ding, Yuqin Pan, Xinqiong Zhang.

Project administration: Yuqin Pan, Xinqiong Zhang.

Resources: Yuqin Pan, Xinqiong Zhang.

Software: Yanan Ding.

Supervision: Yuqin Pan, Xinqiong Zhang.

Validation: Xia Zhao.

Visualization: Xia Zhao, Min Fan.

Writing – original draft: Xia Zhao.

Writing – review & editing: Xia Zhao, Min Fan, Yanan Ding, Yuqin Pan, Xinqiong Zhang.

References

1. Bray F, Laversanne M, Sung H, Ferlay J, Siegel RL, Soerjomataram I, et al. Global cancer statistics 2022: GLOBOCAN estimates of incidence and mortality worldwide for 36 cancers in 185 countries. CA Cancer J Clin. 2024;74(3):229-263.
2. Brust L, Schmidt-Wolf I, Weigl M. The impact of patient engagement on patient safety in care transitions after cancer treatment: Protocol for a systematic review and meta-analysis. PLoS One 2024;19(8):e0307831.
3. Vandraas KF, Reinertsen KV, Kiserud CE, Lie HC. Fear of cancer recurrence among young adult cancer survivors-exploring long-term contributing factors in a large, population-based cohort. J Cancer Surviv 2021;15(4):497-508.
4. Cayrol J, Wakefield CE, Ilbawi A, Donoghoe M, Hoffman R, Echodu M, et al. The lived experience of people affected by cancer: A global cross-sectional survey protocol. PLoS One 2024;19(2):e0294492.
5. Troy AS, Willroth EC, Shallcross AJ, Giuliani NR, Gross JJ, Mauss IB. Psychological Resilience: An Affect-Regulation Framework. Annu Rev Psychol 2023;74:547-576.
6. Egan LA, Park H, Lam J, Gatt JM. Resilience to Stress and Adversity: A Narrative Review of the Role of Positive Affect. Psychol Res Behav Manag 2024;17:2011-2038.
7. Tremblay D, Touati N, Kilpatrick K, Durand MJ, Turcotte A, Prady C, et al. Building resilience in oncology teams: Protocol for a realist evaluation of multiple cases. PLoS One 2022;17(5):e0268393.
8. Zou G, Li Y, Xu R, Li P. Resilience and positive affect contribute to lower cancer-related fatigue among Chinese patients with gastric cancer. J Clin Nurs 2018;27(7-8):e1412-e1418.
9. Ding X, Zhao F, Wang Q, Zhu M, Kan H, Fu E, et al. Effects of interventions for enhancing resilience in cancer patients: A systematic review and network meta-analysis. Clin Psychol Rev 2024;108:102381.
10. Wu X, Xu H, Zhang X, Han S, Ge L, Li X, et al. Self-efficacy, Hope as Mediators Between Positive Coping and Resilience Among Patients With Gastric Cancer Before the First Chemotherapy. Cancer Nurs 2021;44(1):79-85.
11. Ding Y, Zhao X, Zhang X. An intervention based onprotective factors to improve resiience for gastric cancer patients: A pilot study.Indian J Cancer 2023; XX: XX

-XX.
12. Chen W, Xie E, Tian X, Zhang G. Psychometric properties of the Chinese version of the Resilience Scale (RS-14): Preliminary results. PLoS One 2020;15(10):e0241606.
13. Chan KS, Li HC, Chan SW, Lopez V. Herth hope index: psychometric testing of the Chinese version. J Adv Nurs 2012;68(9):2079-85.
14. Dunstan DA, Scott N. Norms for Zung's Self-rating Anxiety Scale. BMC Psychiatry 2020;20(1):90.
15. ZUNG WW. A SELF-RATING DEPRESSION SCALE. Arch Gen Psychiatry 1965;12:63-70.
16. LIU Yang, YANG Jin-giu, YE Ben-lan, SHEN Qu, ZHU Jie-min, CHEN Meiqin. Reliability and validity of the Chinese version of Family Hardiness Index. J Nurs Manag 2014; 14(11),770-772.
17. Xiao SY. The impact of social support on physical and mental health. Chin Ment Health J. 1987 1:183–7.
18. Skivington K, Matthews L, Simpson SA, Craig P, Baird J, Blazeby JM, et al. A new framework for developing and evaluating complex interventions: update of Medical Research Council guidance. BMJ 2021;374:n2061.
19. Beames JR, Lingam R, Boydell K, Calear AL, Torok M, Maston K, et al.Protocol for the process evaluation of a complex intervention delivered in schools to prevent adolescent depression: the Future Proofing Study. BMJ Open 2021;11(1):e042133.
20. Felt JM, Depaoli S, Tiemensma J. Latent Growth Curve Models for Biomarkers of the Stress Response. Front Neurosci 2017;11:315.
21. Zhu S, Sagherian K, Wang Y, Nahm E, Friedmann E. Longitudinal Moderated Mediation Analysis in Parallel Process Latent Growth Curve Modeling in Intervention Studies. Nursing Research 2021;70(3).
22. Damschroder LJ, Aron DC, Keith RE, Kirsh SR, Alexander JA, Lowery JC. Fostering implementation of health services research findings into practice: a consolidated framework for advancing implementation science. Implement Sci 2009;4:50.
23. Glasgow RE, Vogt TM, Boles SM. Evaluating the public health impact of health promotion interventions: the RE-AIM framework. Am J Public Health 1999;89(9):1322-7.
24. Smith JD, Li DH, Rafferty MR. The Implementation Research Logic Model: a method for planning, executing, reporting, and synthesizing implementation projects. Implement Sci 2020;15(1):84.
25. Limbani F, Goudge J, Joshi R, Maar MA, Miranda JJ, Oldenburg B, et al. Process evaluation in the field: global learnings from seven implementation research hypertension projects in low-and middle-income countries. BMC Public Health 2019;19(1):953.

STUDY PERIOD	Before intervention			Intervention period					Post-intervention
TIMEPOINT**	Enrolment	Allocation	Baseline	First chemotherapy phase	Interval phase	Second chemotherapy phase	Interval phase	Third chemotherapy phase	Post-intervention
**ENROLMENT:**									
**Eligibility screen**	X								
**Informed consent**	X								
**Allocation**		X							
**INTERVENTIONS:**									
**Control group:** standard psychological care				X		X		X	
**Intervention group:**									
Fostering trust				X					
Goal setting				X					
Family function					X				
Self-efficacy						X			
Social support							X		
Self-efficacy strengthening								X	
**ASSESSMENTS:**									
**Primary outcome:** levels of resilience			X	X		X		X	
**Secondary outcomes:** levels of hope; levels of negativ emotions; levels of family function; levels of social function; levels of C-reactive protein, cortisol, interleukin			X	X		X		X	
**PROCESS EVALUATION:**									
**Patients and significant others**									
Semi-structured interview									X
Parallel latent growth curve model analysis									X
Goal achievement log book analysis									X
**Intervention staff**									
Semi-structured interview									X

Fig 1. SPIRIT schedule of enrolment, data collection and assessments.

S2 Fig

Click here to access/download;Figure;S2 Fig..tiff

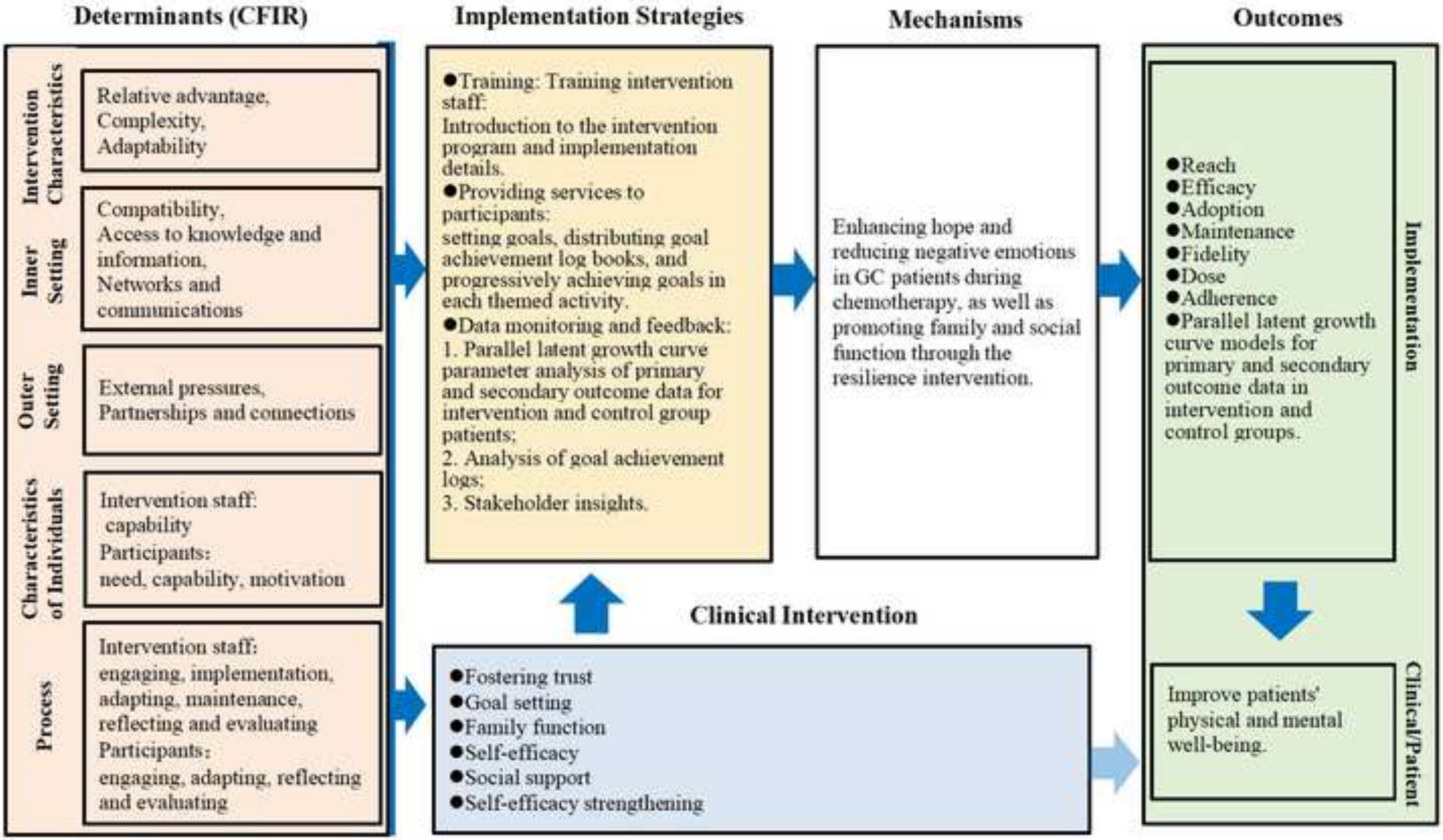

S1 File. Goal achievement log book.

Click here to access/download
Supporting Information
S1 File. Goal achievement log book..docx

S2 File. SPIRIT_Fillable-checklist.

Click here to access/download

Supporting Information

S2 File. SPIRIT_Fillable-checklist..doc

S3 File. Interview guides.

Click here to access/download
Supporting Information
S3 File. Interview guides..docx

S4 File. Approved study protocol by ethics committee.

Click here to access/download

Supporting Information

S4 File. Approved study protocol by ethics committee..pdf
