# Supplementary material for: Complex intervention based on protective factors to improve resilience for gastric cancer patients: Mixed-methods process evaluation protocol
Source: PLoS One. 2025 Aug 13;20(8):e0329834. doi: 10.1371/journal.pone.0329834 (PMC12349701; doi:10.1371/journal.pone.0329834)
Supplement: Attachment — Submitted filename: Response to Reviewers.docx [file pone.0329834.s009.docx]

**List of Responses**

**Dear Editors and Reviewers:**

Thank you for your letter and for the reviewers’ comments concerning our manuscript entitled “Complex intervention based on protective factors to improve resilience for gastric cancer patients: mixed-methods process evaluation protocol” (ID: PONE-D-24-38986). Those comments are all valuable and very helpful for revising and improving our paper, as well as the important guiding significance to our researches. We have studied comments carefully and have made correction which we hope meet with approval. The responses to the comments of the reviewers are presented in blue font, and the revised text and the original modifications are underlined. In response to the suggestions of the reviewers, we have responded to each one below this letter.

Responds to the reviewer’s comments:

**Reviewer #1:**

**Q1:** The manuscript only presents the protocol itself, without providing evidence to verify whether this evaluation method is scientifically sound and efficient. It also lacks clarification on what kind of evaluation outcomes would indicate an effective or ineffective intervention.

**Answer：**Thank you for your suggestion. In response to your query, the manuscript reports a process evaluation protocol embedded within a psychological resilience intervention study for gastric cancer patients undergoing chemotherapy. The purpose of this protocol is to explore the implementation process, mechanisms, influencing factors, and outcomes of the intervention, thereby providing a reference for the development of more comprehensive intervention strategies and broader application. Psychological resilience intervention for gastric cancer patients during chemotherapy is a complex intervention. Process evaluation is an essential component in designing and testing complex interventions, providing scientific evidence and empirical support for the successful implementation and continuous improvement of intervention measures. Therefore, we conducted this process evaluation study to understand the implementation process and the underlying logic of the outcomes. The process evaluation design of this study is guided by the MRC framework, the CFIR framework, and the RE-AIM framework. As reviewed in the relevant literature, the MRC framework is widely used for developing and evaluating complex interventions, the CFIR framework is commonly used to identify factors influencing the implementation of complex interventions, and the RE-AIM framework is one of the most widely used frameworks for planning and evaluating the implementation of complex interventions. Thus, this process evaluation study has a certain degree of scientific rigor and validity. Regarding the "evaluation results of the effectiveness of the intervention" you mentioned, this is the main content of the intervention effect evaluation study, which explores the effectiveness results through the measurement and comparison of outcome indicators before and after the intervention. Since this study is an introduction to the process evaluation protocol, it does not specify what kind of evaluation results indicate the effectiveness or ineffectiveness of the intervention. Thank you for your suggestion. To better clarify the manuscript, the following modifications have been made:

Resilience intervention is complex due to its multifaceted intervention needs across cognitive, emotional, behavioral, and social support dimensions, as well as the dynamic characteristics of individual differences, environmental factors, and psychological states. Therefore, resilience intervention is complex. Complex interventions share two common features: the complexity of the intervention itself and the complexity of its pathways[21]. Process evaluation is a crucial component in designing and testing complex interventions, equally important as outcome evaluation[22]. Process evaluation provides scientific evidence and empirical support for the successful implementation and continuous improvement of intervention measures from many aspects[21]. Therefore, resilience intervention needs to employ process evaluation to identify the mechanisms of each intervention component and the underlying logic behind the outcomes. This study elaborates on the process evaluation protocol for resilience intervention. The protocol aims to understand the implementation process and influencing factors, complementing the outcome evaluation by illustrating the impact of process evaluation on the results, thereby providing insights for developing more refined intervention strategies and broader application.

**Q2:** It is recommended to explain the concept of psychological resilience in the introduction.

**Answer:**Thank you for your recommendation. Based on your suggestion, I have added the following content to the introduction section of the manuscript:

Resilience is a process in which an individual, facing internal and external stress and adversity, activates latent internal cognitions, abilities, or psychological qualities and employs internal and external resources to actively repair and adjust, thereby acquiring the ability, process, or outcome of moving toward positive goals[10].

**Q3:** Since the full text of reference 11 could not be accessed, it is suggested to clarify in the discussion whether the study considered factors such as the classification and staging of gastric cancer, patients’prognoses, and whether the chemotherapy regimens were consistent.

**Answer:**Thank you very much for your valuable and professional suggestions. In response to your concern about the unavailability of the full text of reference 11, I would like to explain that this reference serves as the foundation for the current study. It primarily reports the preliminary application results of the psychological resilience intervention for gastric cancer patients undergoing chemotherapy in this study, demonstrating that the intervention has good feasibility and acceptability. This article is currently under submission, has been accepted and finalized, and is awaiting online publication.

In accordance with your suggestion, I have revised and clarified the discussion section of the manuscript as follows:

The limitations of this protocol lie in the fact that the study only includes patients undergoing chemotherapy after GC surgery, without further considering the impact of factors such as GC classification, staging, and chemotherapy regimens, which may affect the reliability of the study results. Patients in the chemotherapy phase after GC surgery experience physiological stress from surgery and chemotherapy, as well as psychological distress such as anxiety and depression, and are more likely to develop severe psychological problems. The study focuses on this stage, aiming to help patients improve their psychological resilience through targeted psychological interventions. However, due to the limited sample size, further stratification of the patient population may result in subgroups that are too small, thereby affecting the power of statistical analyses and the reliability of the results. In future studies, the sample size will be expanded and more detailed stratifications will be conducted based on patients' different conditions, prognoses, and chemotherapy regimens in a broader population to enhance the representativeness and generalizability of the results.

**Reviewer #2:**

**Q1:** In the introduction you provide an extensive descritpion of a pilot study, to frame your protocol in an existing workflow. I would advise to move this part in a dedicate paragraph of the disucssion. Introduction should focus on framing your work in the actual body of evidence that fostering resilience helps pts with GC and so on.Though you mention many times that this work is parte of MRC framework for complex interventions, it is unclear what the main stream of intervention is. Is this protocol a side work? Is it the core of the bigger intervention?

**Answer：**Thank you very much for your questions and suggestions. The content of the introduction section has been supplemented and modified according to your suggestions, as follows:

The study by Zhang et al. found that individuals with higher resilience are usually able to evaluate themselves more positively and are more proactive in problem-solving and seeking help[12]. A systematic review of resilience in adult cancer patients by Tamura et al. revealed that resilience is closely related to alleviating anxiety and depression in cancer patients and improving their quality of life.

In response to your queries regarding the detailed introduction of the pilot study in the introduction section and the role and relationship of this protocol within the intervention, I would like to explain as follows: The manuscript reports a process evaluation protocol embedded within a psychological resilience intervention study for gastric cancer patients undergoing chemotherapy. The purpose of this protocol is to explore the implementation process, mechanisms, influencing factors, and outcomes of the intervention, thereby providing a reference for the development of more comprehensive intervention strategies and broader application. Therefore, the manuscript provides a detailed introduction to the intervention content in the introduction section as the research premise and basis for this protocol.

Moreover, the psychological resilience intervention for gastric cancer patients during chemotherapy is a complex intervention. Process evaluation is an essential component in designing and testing complex interventions and is equally important as outcome evaluation. It provides scientific evidence and empirical support for the successful implementation and continuous improvement of intervention measures from many aspects. Therefore, we conducted this process evaluation study to understand the implementation process and the underlying logic of the outcomes. The process evaluation design of this study is guided by the MRC framework, the CFIR framework, and the RE-AIM framework.

Thank you for your suggestion. To better clarify the manuscript, the following modifications have been made:

Resilience intervention is complex due to its multifaceted intervention needs across cognitive, emotional, behavioral, and social support dimensions, as well as the dynamic characteristics of individual differences, environmental factors, and psychological states. Therefore, resilience intervention is complex. Complex interventions share two common features: the complexity of the intervention itself and the complexity of its pathways[21]. Process evaluation is a crucial component in designing and testing complex interventions, equally important as outcome evaluation[22]. Process evaluation provides scientific evidence and empirical support for the successful implementation and continuous improvement of intervention measures from many aspects[21]. Therefore, resilience intervention needs to employ process evaluation to identify the mechanisms of each intervention component and the underlying logic behind the outcomes. This study elaborates on the process evaluation protocol for resilience intervention. The protocol aims to understand the implementation process and influencing factors, complementing the outcome evaluation by illustrating the impact of process evaluation on the results, thereby providing insights for developing more refined intervention strategies and broader application.

**Q2:** Some statements are also unclear to me, like those about "positive mindset" of patients. There is a body of literature on hope and resilience that should be accounted for.

**Answer：**Thank you very much for your suggestions. These suggestions can help to further refine the content of the manuscript. In accordance with your suggestions, the concept of psychological resilience and related literature have been supplemented in the manuscript, which can help to understand the expression "positive mindset" of patients. Regarding "hope," it is mentioned in the manuscript as one of the protective factors of psychological resilience. Together with other protective factors, it has been incorporated into the development of the intervention program as a key consideration. Since this manuscript is a report on the process evaluation protocol embedded in the intervention, the introduction section does not provide an extensive discussion. The specific modifications and supplements are as follows:

Resilience is a process in which an individual, facing internal and external stress and adversity, activates latent internal cognitions, abilities, or psychological qualities and employs internal and external resources to actively repair and adjust, thereby acquiring the ability, process, or outcome of moving toward positive goals[10].

The study by Zhang et al. found that individuals with higher resilience are usually able to evaluate themselves more positively and are more proactive in problem-solving and seeking help[12]. A systematic review of resilience in adult cancer patients by Tamura et al. revealed that resilience is closely related to alleviating anxiety and depression in cancer patients and improving their quality of life.

**Q3:**It in unclear whcih pts are you speaking about. Early GC pts? Advanced stage/terminal disease?

**Answer：**Thank you very much for your question. Your query is highly targeted and can help further refine the content of the manuscript. In response to the issue of unclear staging of gastric cancer patients, the discussion section of the manuscript has been supplemented accordingly. The specific content is as follows:

The limitations of this protocol lie in the fact that the study only includes patients undergoing chemotherapy after GC surgery, without further considering the impact of factors such as GC classification, staging, and chemotherapy regimens, which may affect the reliability of the study results. Patients in the chemotherapy phase after GC surgery experience physiological stress from surgery and chemotherapy, as well as psychological distress such as anxiety and depression, and are more likely to develop severe psychological problems. The study focuses on this stage, aiming to help patients improve their psychological resilience through targeted psychological interventions. However, due to the limited sample size, further stratification of the patient population may result in subgroups that are too small, thereby affecting the power of statistical analyses and the reliability of the results. In future studies, the sample size will be expanded and more detailed stratifications will be conducted based on patients' different conditions, prognoses, and chemotherapy regimens in a broader population to enhance the representativeness and generalizability of the results.

**Q4:**The discussion is somehow repetitive of the description of the protocol that you provided in methods section. It should report your previosu reports and those of other authors on the matter and then, maybe, describe how your data and results will contribute. You may also comment on why you chose some framework or methods to collect and analyse data and not other ones.

**Answer：**Thank you very much for your suggestions. The discussion section has been modified accordingly. The specific modifications are as follows:

This study outlines a process evaluation protocol for a complex intervention aimed at enhancing resilience in GC patients during chemotherapy. The protocol explores the implementation process of the intervention to complement and substantiate the results of the effectiveness evaluation. It also revises and refines the intervention program to form a more comprehensive intervention strategy, thereby facilitating the broader application of the intervention among other chronic disease patient populations. Guided by the MRC guidelines[21], CFIR framework[25], and RE-AIM framework[26], the protocol ensures the scientific rigor and completeness of the study. The MRC guidelines are widely used for the development and evaluation of complex interventions. A study by Collet et al.[29] showed that the CFIR framework is a widely used framework to identify facilitators and barriers to the successful implementation of healthcare interventions. A systematic review of RE-AIM utilization by D'Lima et al.[30] indicated that the RE-AIM framework is one of the most widely used frameworks for planning and evaluating the implementation of complex interventions and is now widely used in qualitative and mixed-methods applications. These frameworks will help to fully understand the implementation process of the intervention, the context factors related to the effectiveness of the intervention, and to obtain information to promote better application and dissemination of the intervention among GC patients. Since this intervention is based on resilience itself and its protective factors, and the feasibility and acceptability of the intervention have been demonstrated in GC patients in previous studies, this protocol not only provides strong evidence for the broader application of the intervention in the future but also lays a solid foundation for conducting resilience enhancement studies in other cancer patients and even other chronic disease patients. Our research results will help to develop evidence-based guidelines and provide a scientific basis for health policy and practice. By enhancing patients' resilience, the study aims to improve the overall quality of care for patients and thus bring positive impacts to the healthcare system.

The protocol employs an embedded design, using the IRLM to better understand the core implementation elements of the resilience intervention, including determinants, implementation strategies, mechanisms, outcomes, and potential causal relationships between elements. A mixed-methods design is used to integrate qualitative and quantitative data to comprehensively reflect the implementation of the intervention. A review of process evaluations in randomized controlled trials by French et al.[31] found that of the 31 studies included, 17 used process evaluations, of which nine were quantitative and eight were qualitative. A scoping review of implementation plans for telehealth services by Rangachari et al.[32] showed that most studies (77%) were qualitative or mixed-methods studies aimed at understanding barriers to or facilitators of the intervention. A systematic review of mixed-methods studies by Cardona-Arias et al.[33] pointed out that the use of mixed-methods designs is becoming increasingly common in evaluating the process, outcomes, effectiveness, and implementation of healthcare interventions, enhancing the understanding of causal mechanisms and process feedback, as well as the role of inter-subjective relationships in different interventions. Guided by the CFIR and RE-AIM frameworks and combined with the MRC guidelines, interviews with intervention staff and participants will be conducted to explore potential detailed factors in the implementation of the intervention from qualitative research, which will help to understand the opinions and suggestions of intervention staff on the implementation of the intervention and the views and suggestions of patients on the continued application of the intervention. At the same time, the parallel latent growth curve model will be applied to the quantitative exploration of the mechanisms of impact, and combined with the self-made goal achievement log book, it will further reflect the changes in patients' psychological measurements and the delivery of the intervention during the implementation process with objective data.

The resilience intervention embedded in this protocol is a patient-centered, individualized intervention, that is, targeted interventions are provided according to the different needs and individual circumstances of patients, thereby improving patients' adherence to the intervention and better helping patients enhance their resilience levels. Since the specific intervention content is provided according to the needs of patients, this protocol will reflect the intervention situation through quantitative data measured externally, while conducting interviews with different intervention subjects to understand the real and specific feelings of patients in different situations during the intervention and the common summaries and individual experiences of intervention staff for different intervention objects. The results of this protocol will help to adapt the resilience intervention to the conditions of different cancer patients and even other chronic disease patients and will enhance the sustainability of the intervention in practical applications.

**Q5:**The bibliography is updated but relatively "lean" for a complex work like this.

**Answer：**Thank you very much for your suggestions. The relevant literature has been expanded in the manuscript to support the content.

**Q6:**The overall flow of the paper is not very fluent and I would advise professional, English editing.

**Answer：**Thank you for your suggestion. Your suggestions can help to improve the clarity of the article for readers. The English expression of the manuscript has been polished according to your suggestions.

**Reviewer #3:**

**Q1:** Correct the first-person writing style and replace it with third-person.

**Answer：**Thank you very much for your suggestions. The first-person writing style of the article has been modified to the third-person writing style according to your suggestions.

**Q2:** Correct the location of the quotation numbering, as it does not comply with the Vancouver Standard.

**Answer：**Thank you very much for your suggestions. The location of the references has been modified according to your suggestions.

**Q3:** Since this is a protocol for evaluating an intervention, the quantitative indicators should be more clearly defined (including their formula and expected threshold), as this will facilitate the analysis of the results.

**Answer：**Thank you very much for your questions and suggestions. The manuscript has been supplemented and modified according to your suggestions. The specific modifications are as follows:

At baseline, outcome measures were collected for both groups of patients. The primary outcome measure was the level of resilience, assessed using the 14-Item Resilience Scale[16]. This scale includes two dimensions: personal competence and acceptance of self and life, and uses a Likert 7-point rating scale ranging from 1 (not at all) to 7 (completely), with total scores ranging from 14 to 98 points; higher scores indicate better resilience. Secondary outcome measures included: (1) hope level, assessed using the Herth Hope Index[17], which includes three dimensions: positive attitude towards reality and the future, positive actions taken, and close relationships with others, and uses a Likert 4-point rating scale with total scores ranging from 12 to 48 points (low hope: 12–23 points, moderate hope: 24–35 points, high hope: 36–48 points); (2) negative emotions (anxiety and depression), assessed using the Self-Rating Anxiety Scale and the Self-Rating Depression Scale[18]; (3) family function, assessed using the Chinese Version of the Family Hardiness Index[19], which includes three dimensions: responsibility, control, and challenge, and uses a Likert 4-point rating scale ranging from “strongly disagree” to “strongly agree” (1 to 4 points), with higher scores indicating better family hardiness; (4) social function, assessed using the Social Support Rating Scale[20], which includes three dimensions: objective support, subjective support, and utilization of social support, with higher total scores indicating greater social support; and (5) biochemical indicators, including C-reactive protein, cortisol, and interleukin levels, obtained through the hospital database.

**Q4:** It is suggested that the patient inclusion criteria be included.

**Answer：**Thank you very much for your questions and suggestions. The inclusion, exclusion, and dropout criteria for patients have been supplemented. The specific modifications are as follows:

Inclusion criteria were as follows (1) patients with a histopathological diagnosis of gastric cancer, (2) age ≥ 18 years, (3) post-gastrectomy chemotherapy planned to be implemented at the study hospital, (4) patients with a permissible medical condition who provided informed consent and were able to cooperate, (5) patients with an estimated survival of more than 6 months and eligible for follow-up, (6) patients who completed the resilience intervention. Exclusion criteria included (1) patients with communication or comprehension barriers, (2) patients with other severe comorbidities, (3) patients with a history of psychological disorders or psychiatric illness, (4) patients participating in other clinical studies. Dropout criteria were applied to patients who did not complete the entire resilience intervention for any reason.

**Q5:** It is suggested that details be provided on how the questionnaires assessing resilience, depression, social support, and other factors will be administered.

**Answer：**Thank you very much for your questions and suggestions. The detailed information of the questionnaires has been supplemented according to your suggestions. The specific supplements are as follows:

At baseline, outcome measures were collected for both groups of patients. The primary outcome measure was the level of resilience, assessed using the 14-Item Resilience Scale[16]. This scale includes two dimensions: personal competence and acceptance of self and life, and uses a Likert 7-point rating scale ranging from 1 (not at all) to 7 (completely), with total scores ranging from 14 to 98 points; higher scores indicate better resilience. Secondary outcome measures included: (1) hope level, assessed using the Herth Hope Index[17], which includes three dimensions: positive attitude towards reality and the future, positive actions taken, and close relationships with others, and uses a Likert 4-point rating scale with total scores ranging from 12 to 48 points (low hope: 12–23 points, moderate hope: 24–35 points, high hope: 36–48 points); (2) negative emotions (anxiety and depression), assessed using the Self-Rating Anxiety Scale and the Self-Rating Depression Scale[18]; (3) family function, assessed using the Chinese Version of the Family Hardiness Index[19], which includes three dimensions: responsibility, control, and challenge, and uses a Likert 4-point rating scale ranging from “strongly disagree” to “strongly agree” (1 to 4 points), with higher scores indicating better family hardiness; (4) social function, assessed using the Social Support Rating Scale[20], which includes three dimensions: objective support, subjective support, and utilization of social support, with higher total scores indicating greater social support; and (5) biochemical indicators, including C-reactive protein, cortisol, and interleukin levels, obtained through the hospital database.

**Q6:** In the semi-structured interview guidelines, it is suggested that the targeted area be indicated (reach, efficacy, adoption, and maintenance); a table could be included given its importance.

**Answer：**Thank you very much for your questions and suggestions. The table has been supplemented in Supplementary File 3 according to your suggestions. The specific supplements are as follows:

The domains and content of the interview guide.

| Interviewee | Target domain | Theoretical framework | Interview content |
| --- | --- | --- | --- |
| Intervention staff | Perspectives on the intervention |  | - How do you understand our intervention plan? |
|  | Impact factors of intervention implementation | CFIR-intervention characteristics | - In your opinion, what are the advantages of the intervention measures in this study compared to routine psychological care and other psychological care methods? - What do you perceive as the complexities in implementing the intervention? - Is our intervention adaptable to different recipients and environments? |
|  |  | CFIR-inner setting | - How does this intervention align with our existing psychological care models and values? How does it fit with patients' values? How does it integrate into our routine psychological care processes and environment? - Can you tell me about your approach to building relationships with patients? - Could you describe efforts you made during past intervention implementations to better understand the needs of patients during the intervention process? |
|  |  | CFIR-outer setting | - What key partners does our organization have in advancing intervention implementation? How do these partnerships facilitate or hinder the adoption and implementation of the intervention? - Have there been pressures from other stakeholders and the work environment (clinical nurses)? How do they influence the implementation of the intervention? |
|  |  | CFIR-characteristics of individuals | - What efforts did you make to implement the intervention? Do you feel confident in implementing the intervention effectively? |
|  |  | CFIR-process | - How do you ensure that the intervention is implemented correctly and comprehensively? - What challenges did you encounter during the implementation process? - What improvements are needed for the intervention to better meet the needs of recipients and adapt to external implementation environments? - How do we ensure the sustainability of the intervention? - Do you have any additional thoughts or experiences to share regarding the implementation of this intervention? |
| Participants | Perspectives on the intervention |  | - Could you please share your experience participating in this service activity? - What were the most important activities you engaged in throughout the psychological care process? (Which part do you feel helped the most?) Were there any activities that were unnecessary or did not yield the expected results? - How did your physical and mental state fluctuate throughout the psychological care process? |
|  | Reach | RE-AIM | - What motivated you to participate in this activity? Why did you join this activity? |
|  | Efficacy | RE-AIM | - What impact did the psychological care service have on key outcomes? (e.g., impact on physical and mental states, or on family) |
|  | Adoption | RE-AIM | - Are you still satisfied with our psychological care service? |
|  | Maintenance | RE-AIM | - Do you think this psychological care service is necessary? (Is there a need for its existence and expansion?) Do you believe this psychological care service should continue to be provided? Will you continue the psychological exercise methods in the activities after the end? |
|  | Impact factors of intervention implementation | CFIR-intervention characteristics | - In your opinion, what are the advantages of this new care approach compared to the psychological care or other psychological services you have received before? - Do you find this new psychological care approach complex compared to previous experiences? - What adjustments did you make to meet our task requirements? |
|  |  | CFIR-inner setting | - How well does our psychological care align with your values and established beliefs? Are there any differences? - Do you believe the knowledge provided in our psychological care is what you need? Are there any knowledge areas we did not cover that you would find helpful? - What are your thoughts on our combined online and offline approach? |
|  |  | CFIR-outer setting | - Did you face any external pressures or challenges while completing our task requirements? - How did you maintain contact with our intervention personnel during the psychological care process? How was the relationship? |
|  |  | CFIR-characteristics of individuals | - Do you feel this psychological care arrangement meets your needs? - Were you actively involved in the intervention? How motivated were you to participate? - What efforts did you make to complete our tasks? Do you feel confident in your ability to complete tasks well? |
|  |  | CFIR-process | - What challenges did you encounter in completing the psychological care tasks? - Do you think this type of psychological care needs adjustments based on your specific circumstances? - Is there any challenge or experience we haven't covered that you would like to share with me? |

**Q7:** Something very important is that reference No. 11 cannot be found. Furthermore, it is incomplete. This makes access to the results of the pilot study impossible.

**Answer：**Thank you very much for your valuable and professional suggestions. In response to your concern about the unavailability of the full text of reference 11, I would like to explain that this reference serves as the foundation for the current study. It primarily reports the preliminary application results of the psychological resilience intervention for gastric cancer patients undergoing chemotherapy in this study, demonstrating that the intervention has good feasibility and acceptability. This article is currently under submission, has been accepted and finalized, and is awaiting online publication.

**Q8:** There are other corrections that are highlighted in yellow with a note.

**Answer：**

Thank you very much for your suggestions. The content of the manuscript has been revised according to your comments.

**Reviewer #4:**

**Q1:** The study demonstrates limited novelty.

**Answer：**We sincerely appreciate your review of our study and the valuable suggestions you have offered. In response to your comment that "The study demonstrates limited novelty," we understand your perspective and would like to further elaborate on the innovative aspects of our research and its significant contributions to the existing body of literature.

This manuscript reports a process evaluation protocol for a complex intervention aimed at enhancing psychological resilience in gastric cancer patients undergoing chemotherapy. The psychological resilience intervention for gastric cancer patients during chemotherapy on which this study is based not only incorporates psychological and behavioral change techniques but also specifically focuses on the psychological needs of patients during chemotherapy. By identifying and strengthening protective factors such as self-efficacy, hope, family function, and social support, it offers a comprehensive psychological support strategy for gastric cancer patients, which is relatively unique in the existing research.

Moreover, this manuscript pays attention to the implementation process of intervention research. Through process evaluation, it thoroughly explores the implementation process, influencing mechanisms, and contextual factors of the intervention measures. This comprehensive process evaluation approach can provide valuable implementation experience and directions for improvement for future research.

Additionally, we have adopted the MRC framework, the CFIR framework, and the RE-AIM framework. These frameworks provide a systematic method for evaluating the implementation process of complex interventions, enabling us to more scientifically assess the fidelity, dose, adherence, and influencing factors of the intervention measures, thereby providing theoretical support for the optimization of the intervention.

At the same time, our study emphasizes the importance of a patient-centered approach. By incorporating feedback from patients and healthcare providers, we ensure the feasibility and acceptability of the intervention measures. This patient-centered design not only enhances the effectiveness of the intervention but also strengthens its sustainability in practical applications.

We firmly believe that our study demonstrates significant innovation in its design and implementation. It offers new strategies for psychological support for gastric cancer patients and provides valuable references and scientific basis for psychological resilience research in other chronic disease patients as well as for health policy and practice.

We look forward to making a positive contribution to improving patients' psychological states and quality of life through our research. Thank you once again for your response and review of our study. We hope that the above statement helps you better understand the content of our manuscript and discover the highlights of our research.

**Q2:** The description of intervention details is inadequate, as it neither explains how to prevent contamination among subjects due to interactions, nor how to avoid selection bias and standardize the research protocol.

**Answer：**Thank you very much for your questions and suggestions. Your observation that the description of the intervention details is not sufficient is very pertinent, and we fully understand and attach great importance to this point. Below is our detailed response to your comments:

Regarding your concern about "no explanation on how to prevent interaction contamination between participants," the manuscript mentions that patients were grouped according to their admission order, with a one-month interval between the inclusion times of the two groups. "Following patient informed consent, those admitted from August 2023 to March 2024 were assigned to the control group, while those admitted from April 2024 to December 2024 were assigned to the intervention group." This method of grouping can avoid contamination issues between the two groups in terms of time and space.

In response to the question of "how to avoid selection bias and standardize the research protocol," due to ethical and practical reasons, we used convenience sampling to recruit patients and implemented single blinding for the outcome assessors. In addition, when recruiting participants, we tried to ensure the diversity and representativeness of the sample. Secondly, during the data analysis phase, we adopted an intention-to-treat analysis method, including all randomly assigned participants in the final analysis, regardless of whether they completed the entire intervention process. This approach helps to reduce selection bias caused by loss to follow-up or withdrawal from the study.

To ensure the standardization of the research protocol, we took the following measures: Before the intervention, the intervention developers trained the research team members and clinical nurses, all of whom held psychological counseling certification. They provided a detailed introduction to the intervention plan and emphasized important considerations for its implementation, ensuring that they had a clear understanding of the intervention measures and could execute them consistently, thereby ensuring the standardization and homogeneity of the intervention implementation. At the same time, the intervention was delivered through face-to-face communication and WeChat for real-time supervision of the patients, with dynamic adjustment of the intervention plan to ensure high-quality implementation. Finally, we established a strict data quality control system, including standardized processes for data collection, entry, and analysis. All data were entered by two individuals to ensure the accuracy and completeness of the data.

For detailed information on the intervention, please refer to reference 11, which serves as the basis for this study. It mainly reports the preliminary application results of the psychological resilience intervention for gastric cancer patients undergoing chemotherapy in this study. This article is currently under submission, has been accepted and finalized, and is awaiting online publication.

In accordance with the reviewer's suggestions, we have made modifications and supplements in the corresponding parts of the manuscript. The specific modifications and supplements are as follows:

An intention-to-treat analysis will be employed to provide a more comprehensive and robust assessment of the intervention effects, with multiple imputations for missing values conducted using R software.

**Q3:** The manuscript fails to thoroughly detail the methods, steps, and scientific rationale of the intervention scheme, thus necessitating clarification on its development process, theoretical basis, and supportive evidence.

**Answer：**Thank you very much for your questions and suggestions. In response to your queries, I would like to explain that this manuscript reports a process evaluation protocol embedded within a psychological resilience intervention study for gastric cancer patients undergoing chemotherapy. The purpose of this protocol is to explore the implementation process, mechanisms, influencing factors, and outcomes of the intervention, thereby providing a reference for the development of more comprehensive intervention strategies and broader application. The development process of the intervention program serves as the basis for the preliminary research, and this study focuses on exploring the implementation process of the intervention. Therefore, the methods, steps, and scientific principles of the intervention program are not elaborated in detail.

**Q4:** There is a lack of a comprehensive discussion on quality control during the intervention process, covering aspects such as personnel, subject compliance, and data analysis, so the authors must provide a detailed account of quality control strategies for each stage to ensure the validity of the results and protocol.

**Answer：**Thank you very much for your questions and suggestions. The content related to the quality control of the intervention has been supplemented in the manuscript. The specific content is as follows:

**Quality Control**

**Study design phase:**

(1) A mixed-methods approach will be used, integrating quantitative and qualitative data to comprehensively explore the implementation process of the intervention, ensuring the accuracy and comprehensiveness of the results.

(2) The mixed-methods study will be guided by the MRC guidelines, the RE-AIM framework, the CFIR framework, and the parallel latent growth curve model to ensure the scientific rigor and reliability of the process evaluation.

**Intervention implementation phase:**

(1) Before the intervention, the intervention developers will train the research team members and clinical nurses, providing a detailed introduction to the intervention plan and emphasizing important considerations for its implementation, ensuring that they have a clear understanding of the intervention measures and can execute them consistently, thereby ensuring the standardization and homogeneity of the intervention implementation.

(2) Intervention staff: All intervention implementers will hold psychological counseling certification. The research team will hold weekly discussion meetings to report on the progress of the intervention and collectively discuss and resolve any issues that arise, further ensuring the quality of the intervention implementation.

(3) Intervention subjects and their families: The purpose, significance, and potential benefits of the intervention will be explained in detail to the patients and their families. Their consent will be obtained, and they will be asked to sign an informed consent form. Family members will be involved throughout the process and encouraged to provide supervision, enhancing the enthusiasm and compliance of the study subjects.

(4) Intervention implementation: The intervention will be delivered through face-to-face communication and WeChat for real-time supervision of the patients, with timely resolution of any intervention-related issues. The intervention plan will be dynamically adjusted to ensure high-quality implementation.

**Data collection phase:**

(1) Quantitative data: The final data will be formed through a combination of self-assessment by patients via the goal achievement log book and assessments recorded by researchers, providing a multi-perspective reflection of the intervention implementation. Qualitative data: The interview guide will be developed based on the question repository provided by the CFIR website and refined after a pilot interview. The data collection tools will be scientifically reliable. Semi-structured interviews will be conducted by experienced interviewers to facilitate comprehensive expression of patients' thoughts and ensure the relative objectivity of the interview data.

(2) Throughout the data collection process, investigators will be well-versed in the content, scoring methods, and usage notes of each scale, using a unified set of instructions to personally guide patients in filling out the forms, reducing information bias. If patients are unable to complete the questionnaire independently, researchers will read out the questionnaire content and fill it in according to the patients' responses, ensuring the authenticity of the questionnaire completion. After the questionnaires are collected, they will be promptly checked for any missing items and completed as necessary to ensure data integrity.

(3) Data processing will be conducted by professionals who will double-check and enter the data to ensure the objectivity and accuracy of data processing.

**Data analysis phase:**

Data processing and analysis will be conducted by external personnel independent of the intervention implementation process to achieve single-blinding and reduce bias in result analysis.

**Reviewer #5:**

**Q1:** Clarifying sentence 2 in the introduction in which the authors describe severe psychological distress with examples and psychological challenges, without examples. I would recommend either providing examples of psychological challenges as the authors did with severe psychological distress, or simply provide definitions so the readers understand the difference.

**Answer：**Thank you very much for your questions and suggestions. The corresponding parts of the manuscript have been revised. The specific modifications are as follows:

Study has shown that chemotherapy is an important therapeutic modality for GC patients, but it is also a significant source of physical and psychological stress[5]. In a study on the risk factors for the efficacy of chemotherapy in advanced GC patients conducted by Zhang et al.[6], psychological distress was identified as an important factor that affects patients' physical symptoms and treatment outcomes.

**Q2:** To elaborate further on how participants were chosen for the control and intervention group. As its written, it appears, its based on when patients were admitted, which would be challenging for other researchers to replicate the study. Perhaps there were more criteria or some sort of additional randomization methodology that determined where participants were placed in the study; please clarify.

**Answer：**Thank you very much for your valuable suggestions. We understand your concerns regarding the potential impact of the grouping method on the reproducibility of the study. Below is our detailed response to this issue:The study chose to group patients according to their admission order based on ethical and practical considerations. By using admission time, patients who met the inclusion and exclusion criteria during the same time period were assigned to the control or intervention group, with a one-month washout period between the two groups to avoid temporal and spatial contamination. This approach mitigates selection bias to some extent and ensures the transparency and fairness of the grouping process. It also avoids the ethical issue of including eligible patients in different groups within the same time frame. Moreover, grouping based on admission order ensures that the intervention can be implemented in a timely manner without delaying the intervention due to randomization waiting periods. Additionally, given the two-month intervention period, a blended approach combining online and offline methods was used to ensure continuous and complete delivery of the intervention content while reducing time costs. The intervention site is a top-tier hospital, which ensures an adequate sample size of cancer patients undergoing chemotherapy. Therefore, grouping patients according to their admission order is feasible and reproducible in this study.

We fully understand the potential limitations of grouping based on admission order. Therefore, we have discussed this limitation in detail in the discussion section and proposed suggestions for future studies. The specific modifications are as follows:

Additionally, due to ethical and practical reasons, the resilience intervention adopted a non-concurrent quasi-experimental design, with patients assigned to groups based on their admission order. This method does not achieve ideal randomization and may affect the scientific rigor and reliability of the study. However, by using admission time to assign patients who meet the inclusion and exclusion criteria to the control or intervention group, with a one-month washout period between the two groups to avoid temporal and spatial contamination, selection bias can be mitigated to some extent and the transparency and fairness of the grouping process can be ensured. This approach also avoids the ethical issue of including eligible patients in different groups within the same time frame. Moreover, grouping based on admission order ensures that the intervention can be implemented in a timely manner without delaying the intervention due to randomization waiting periods. Additionally, given the two-month intervention period, a blended approach combining online and offline methods is used to ensure continuous and complete delivery of the intervention content while reducing time costs. Therefore, in future studies, the research team will consider expanding the scope of intervention subjects, adopting a multi-center design, and combining randomization methods to further enhance the scientific rigor, reproducibility, and reliability of the study results.

**Reviewer #6:**

**Q1:** Given that the manuscript lacks actual results and primarily outlines a methodology—resembling a grant proposal—I do not recommend it for publication in *PLOS ONE*.

**Answer：**Thank you very much for your in-depth review and valuable suggestions. We greatly appreciate your feedback and have carefully considered and discussed your recommendations. Below are our responses and explanations:Regarding the nature of the study and the suitability of the publication platform: We fully understand your point that our manuscript resembles a funding application rather than a research article containing actual results. However, this study reports a process evaluation protocol for a complex intervention aimed at enhancing psychological resilience in gastric cancer patients undergoing chemotherapy. The protocol explores the implementation process of the intervention, thereby complementing and substantiating the results of the effectiveness evaluation. It also revises and refines the intervention program to form a more comprehensive intervention strategy, facilitating broader application among other chronic disease patient populations. Although the final results have not yet been collected, our study protocol has been prospectively registered, obtained ethical approval, and has commenced implementation. We plan to publish the actual results in a subsequent article after the study is completed. In the current manuscript, we have chosen to focus on the study design and methods to ensure that readers can clearly understand our research plan and expected objectives. We believe that by detailing the study design and methods, we can provide valuable references for other researchers and lay a solid theoretical foundation for future research results. *PLOS ONE* is an open-access journal covering a wide range of disciplines, with the mission of publishing all peer-reviewed scientific and medical research, regardless of whether it contains final results. We chose to publish in *PLOS ONE* because we believe that our study protocol and methods have significant scientific value and can provide useful information to researchers in related fields. Moreover, *PLOS ONE* accepts study protocols and methodological articles, which aligns with the nature of our research.。

Special thanks to you for your good comments.

We tried our best to improve the manuscript and made some changes in the manuscript. We appreciate for Editors/Reviewers’ warm work earnestly, and hope that the correction will meet with approval.

Once again, thank you very much for your comments and suggestions.

Best regards.

Xia Zhao

School of Nursing, Anhui Medical University, Hefei, Anhui, China.

E-mail: [2635370481@qq.com](mailto:2635370481@qq.com)

July 11, 2025
